# First Report of *Aleurocanthus spiniferus* on *Ailanthus altissima*: Profiling of the Insect Microbiome and MicroRNAs

**DOI:** 10.3390/insects11030161

**Published:** 2020-03-03

**Authors:** Giovanni Bubici, Maria Isabella Prigigallo, Francesca Garganese, Francesco Nugnes, Maurice Jansen, Francesco Porcelli

**Affiliations:** 1 Istituto per la Protezione Sostenibile delle Piante, Consiglio Nazionale delle Ricerche, via Amendola 165/A, 70126 Bari, Italy; mariaisabella.prigigallo@ipsp.cnr.it; 2 Dipartimento di Scienze del Suolo, della Pianta e degli Alimenti, Università degli Studi di Bari Aldo Moro, via Amendola 165/A, 70126 Bari, Italy; francesca.garganese@uniba.it (F.G.); francesco.porcelli@uniba.it (F.P.); 3 Istituto per la Protezione Sostenibile delle Piante, Consiglio Nazionale delle Ricerche, via Università 133, 80055 Portici, Italy; francesco.nugnes@ipsp.cnr.it; 4 Ministry of Agriculture, Nature and Food Quality, Laboratories Division, Netherlands Food and Consumer Product Safety Authority (NVWA), Geertjesweg 15, 6706 EA Wageningen, The Netherlands; m.g.m.jansen@nvwa.nl

**Keywords:** spiny blackfly, orange spiny whitefly, tree of heaven, alien, invasive or quarantine pest

## Abstract

We report the first occurrence of the orange spiny whitefly (*Aleurocanthus spiniferus*; OSW) on the tree of heaven (*Ailanthus altissima*) in Bari, Apulia region, Italy. After our first observation in 2016, the infestation recurred regularly during the following years and expanded to the neighboring trees. Since then, we have also found the insect on numerous patches of the tree of heaven and other plant species in the Bari province. Nevertheless, the tree of heaven was not particularly threatened by the insect, so that a possible contribution by OSW for the control of such an invasive plant cannot be hypothesized hitherto. This work was also aimed at profiling the microbiome of OSW feeding on *A. altissima*. For this purpose, we used the denaturing gradient gel electrophoresis (DGGE) and the deep sequencing of small RNAs (sRNAs). Both techniques unveiled the presence of “*Candidatus* Portiera” (primary endosymbiont), *Wolbachia* sp. and *Rickettsia* sp., endosymbionts already reported for other Aleyrodidae. Deep sequencing data were analyzed by four computational pipelines in order to understand the reliability of the detection of fungi, bacteria, and viruses: Kraken, Kaiju, Velvet, and VelvetOptimiser. Some contigs assembled by Velvet or VelvetOptimiser were associated with insects, but not necessarily in the *Aleurocanthus* genus or Aleyrodidae family, suggesting the non-specificity of sRNAs or possible traces of parasitoids in the sample (e.g., *Eretmocerus* sp.). Finally, deep sequencing data were used to describe the microtranscriptome of OSW: 56 canonical and at least four high-confidence novel microRNAs (miRNAs) were identified. The overall miRNA abundance in OSW was in agreement with previous works on *Bemisia tabaci*, and bantam-3p, miR-276a-3p, miR-317-3p, miR-750-3p, and mir-8-3p were the most represented miRNAs.

## 1. Introduction

The orange spiny whitefly (OSW), *Aleurocanthus spiniferus* (Quaintance, 1903) originated in tropical Asia and has spread to Africa, Australia, and the Pacific Islands. In Europe, it was reported for the first time in Italy [1], and it is spreading all over the Mediterranean Countries [2,3]. *Aleurocanthus* Quaintance and Baker is a paleotropical genus, currently including about 80 described species [4]. OSW is listed as a quarantine pest in Europe and it is now included in the EU Annex II/A1 (from December 2020 it will be included in EU 2016/2031 Annex II/part B) as well as in the EPPO A2 list [5]. It has been reported to infest about 90 plant species of 38 plant botanical families [6]. *Citrus* spp. L. are the main economically important hosts, and grapes (*Vitis vinifera* L.), guavas (*Psidium guajava* L.), pears (*Pyrus* spp. L.), persimmons (*Diospyros kaki* L. f.), and roses (*Rosa* spp. L.) are commonly infested as well [6,7].

The tree of heaven [*Ailanthus altissima* (Mill.) Swingle] is a deciduous tree in the family Simaroubaceae, native to China and Taiwan (http://www.plantsoftheworldonline.org). The tree grows rapidly, has a remarkable suckering ability, and suppresses competition with allelopathic chemicals that make it a noxious weed and vigorous invasive species in Australia, the United States of America, New Zealand, and Europe [8]. In several European countries, it is officially regulated or listed as an invasive alien plant [9]. A number of pathogens and pests have been reported on *A. altissima*: 46 phytophagous arthropods, 16 fungi, and one potyvirus have been reported from its native areal, China, whereas nine insect herbivores and 68 fungi have been reported from North America, Europe, and Asia. However, only a few of them have been considered as major threats, e.g., *Verticillium albo-atrum* Reinke and Berthold, *V. nonalfalfae* Inderbitzin et al. [10,11], *Lycorma delicatula* (White) (Hemiptera: Fulgoridae) [9], two weevils, *Eucryptorrhynchus brandti* (Harold) and *E. chinensis* Olivier (Coleoptera: Curculionidae), and two lepidopteran species, *Samia cynthia* Drury (Lepidoptera: Saturnidae) and *Eligma narcissus* Cramer (Lepidoptera: Nolidae) [12]. Due to the damage caused on the tree, these pests have gained much attention for the evaluation of their potential in the biological control of the tree of heaven [12,13].

**Figure 1:**
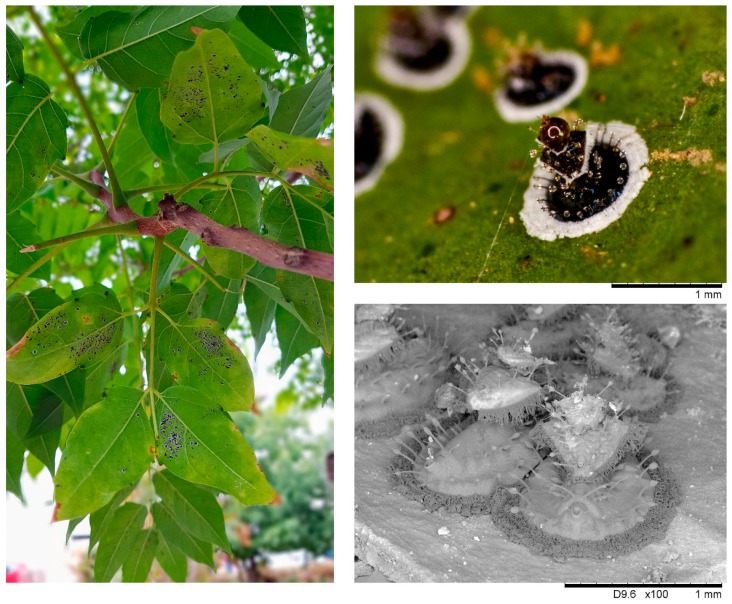
*Aleurocanthus spiniferus* on *Ailanthus altissima* leaves in Bari, Apulia region, Italy. Puparia on the underside of the leaves (on the left). Close-up of puparia taken by a photo camera (top right) and a scanning electron microscope (bottom right).

In July 2016, we observed a heavy insect infestation on a few trees of heaven located in the university campus of Bari, Apulia region, Italy (Figure 1). The insect was identified as OSW (Hemiptera: Aleyrodidae) according to Porcelli [1] and Jansen and Porcelli [4]. Very likely, the insect moved or was driven to the trees of heaven from the neighboring citrus trees, which are among their main hosts [6]. *Ailanthus altissima* infested shoots showed growth stunting with chlorotic leaves, which developed necrotic areas and eventually shed prematurely in the early summer. The insect demes were covered by plenty of honeydew drops. The infestation recurred regularly and expanded during the following years across the Bari province but with lower population densities in comparison with the first observed.

It is known that Aleyrodidae species harbor endocytobiont Bacteroides playing fundamental roles for their fitness. Also, it has been demonstrated that endosymbionts can both protect their host against enemies (i.e., parasitoids, other bacteria, and viruses) and influence some biological traits (e.g., fitness, sex ratio, and host range) usually ameliorating the growth rate of the host under certain stress conditions [14,15]. Although OSW has a wide host range, we hypothesized that shifting to a new host, *A. altissima*, could be also associated with some extent to a new assembly of endosymbionts.

Therefore, we aimed at describing the microbiome of that OSW population and, for this purpose, we carried out the deep sequencing of small RNAs (sRNAs), which allowed us to identify in one assay bacteria, fungi, viruses, and insects associated with OSW. In fact, sRNAs are produced in all these organisms [16], thus they can be used to track their presence in a microbiome. In addition, sRNA-Seq allows the identification and quantification of microRNAs (miRNAs), a class of non-coding RNA molecules (ca. 18–24 nucleotides or nt) found in plants, animals and some viruses, that functions in RNA silencing and post-transcriptional regulation of gene expression [17].

The RNA sequencing has not been used so far in insects to detect at the same time both microorganisms and viruses, but several other techniques, including the polymerase chain reaction-denaturing gradient gel electrophoresis (PCR-DGGE) [18,19,20,21] and the next-generation sequencing (NGS) [22,23,24,25,26,27,28], have been adopted to investigate particular taxa.

In this work, we documented the first occurrence of OSW on *A. altissima* and used sRNA deep sequencing to obtain the microbiome profile and sRNA transcriptome.

## 2. Materials and Methods

### 2.1. Insect Identification

OSW puparia were collected yearly since July 2016 from diverse host plants, including the tree of heaven, in the Bari province. More than one hundred puparia were slide-mounted after discarding the layering previous instar exuvia [29]. The slide mounting procedure (https://zenodo.org/record/3471649#.Xk1cr2hKhPY) made the puparia cuticle clear enough to discriminate relevant details. Thus, the mounted puparia were scrutinized and identified using a compound light microscope to study taxonomic characters [1,4].

### 2.2. Denaturing Gradient Gel Electrophoresis (DGGE)

Before DNA extraction, 10 single specimens of OSW infesting *A. altissima* were surface sterilized in 70% ethanol and rinsed three times in sterile distilled water. DNA was extracted with a slight modification of the protocol described by Gebiola et al. [30], where specimens were homogenized with a sterile pestle directly in the extraction buffer (Chelex 100 + pK). The V3 hypervariable region of 16S rDNA was amplified by PCR [31,32] and the products were analyzed by a slight modification of the DGGE technique described by Muyzer et al. [33] using a 40%–65% denaturing gradient (90 V for 20 h). Known endosymbionts such as *Wolbachia* Hertig from *Encarsia formosa* Gahan, *Cardinium* Zchori-Fein et al. from *Encarsia pergandiella* Howard, *Rickettsia* da Rocha-Lima from *Pnigalio soemius* (Walker), *Spiroplasma* Saglio et al. from *Drosophila neotestacea* Grimaldi, James and Jaenike, and *Arsenophonus* Gherna et al. from *Bemisia tabaci* (Gennadius) were used as positive controls. All dominant DGGE bands were excised, eluted, used as templates for PCR with the primers 341f/518r, and directly sequenced. Obtained sequences were blasted against the GenBank database.

### 2.3. Deep Sequencing of Small RNAs

Total RNA was extracted from 100 mg of OSW puparia using TRIzol™ (Thermo Fisher Scientific, Bedford, MA, USA), according to the manufacturer’s instructions, subjected to DNase digestion using TURBO DNA-free™ Kit (Thermo Fisher Scientific, Bedford, MA, USA), and suspended in nuclease-free water. OSW puparia were rinsed three times in sterile distilled water before RNA extraction. One microgram RNA was sent to IGA Technology Services (Udine, Italy) for library preparation and sequencing. The TruSeq Small RNA Library Prep Kit (Illumina, San Diego, CA, USA) was used for library construction, and 50 bp reads were sequenced on HiSeq 2500 system (Illumina, San Diego, CA, USA) in single-end mode.

The sequencing raw reads were subjected to adapter removal, quality control and filtering using FASTX-toolkit version 0.1 [34], FASTQC version 0.72+ (http://www.bioinformatics.babraham.ac.uk/projects/fastqc/), and Filter FASTQ tool version 1.1.1 [35], respectively, in order to obtain high-quality reads of 15–35 nt to be used for the downstream analysis. These steps were performed within a locally-installed Galaxy version 19.01 [36].

### 2.4. NGS Data Analysis

#### 2.4.1. Microbiomics

High-quality sequencing reads of 15–35 nt were used to infer the complete microbiome of OSW, i.e., the presence of reads associated with insects, fungi, bacteria, and viruses. Since the taxonomic assignment of sequencing reads might be affected by the bioinformatics approach, including the reference database, we used four computational pipelines and compared their results: Kraken, Kaiju, Velvet, and VelvetOptimiser (the latter two tools were followed by a blastn search). Kraken is a taxonomic sequence classifier that assigns taxonomic labels to short DNA reads [37]. We subjected the high-quality sequencing reads to version 1.2.4 of this tool within the public Galaxy server (https://usegalaxy.org/), where it utilizes databases for bacteria, fungi or plasmids. Kaiju is a web-based program (http://kaiju.binf.ku.dk/) for taxonomic classification of sequencing reads from metagenomic whole genome sequencing or metatranscriptomics experiments [38]. We used version 1.7.2. with default parameters and the NCBI nr database including non-redundant proteins of bacteria, archaea, viruses, fungi, and microbial eukaryotes. Velvet 1.2.10.0 was used to assemble reads into contigs with a hash length of 21 [39]. The hash length value affects the number and length of contigs finally obtained: smaller hash lengths yield more contigs, but shorter in size, whereas higher hash lengths yield fewer contigs, but longer. VelvetOptimiser assembles reads using a range of hash length values in order to maximize the contig length, contig number, N50 value or other parameters as specified by the user [40]. We used this tool with a range of hash length between 19 and 31. Contigs assembled with Velvet or VelvetOptimiser were then used as queries for the search against NCBI BLAST nucleotide collection database (nr/nt) using the blastn algorithm on the web service. Only BLAST hits with more than 95% sequence similarity and e-value lower than 10^−5^ were retained.

The outputs of these four pipelines were processed with MEGAN6 for the graphical representations [41].

#### 2.4.2. Classification of Small RNAs

In order to have an overview of the sRNA features in OSW, we carried out several analyses. Length distribution of reads was extracted from the FASTQC output, imported into Microsoft Excel Professional 2016 and used to generate histograms. This procedure was done both for redundant and non-redundant reads, the latter obtained using Collapse sequences tool version 1.0.1 [34] within the locally-installed Galaxy.

Sequencing reads were used as queries to search sequence similarities in the Rfam database, separately by RNA categories such as rRNAs, miRNAs, several classes of repetitive elements, transcripts and other Rfam families. Alignment to the *B. tabaci* genome using bowtie 2 with default parameters [42] was used to determine the number of reads matching the genome. *B. tabaci* isolate MEAM1 (assembly ASM185493v1; 19,751 scaffolds) was used because it is a species in the same family (Aleyrodidae) of OSW, which genome is not yet available. The remaining reads were considered as unassigned RNA. The number of reads associated with these features was used to generate pie charts by Microsoft Excel Professional 2016.

#### 2.4.3. Identification of Known and Novel MicroRNAs Including IsomiRs

miRDeep2 was used to identify miRNAs in OSW [43]. As inputs to this tool, we submitted the sequencing reads and the genome of *B. tabaci* isolate MEAM1 (assembly ASM185493v1), without known miRNA and precursor sequences. MiRDeep2 predicts precursors and miRNAs in a genome and associates them with a 0–10 score and a randfold significance. No arbitrary threshold was used to select sequences, and all predicted miRNAs were retained. MicroRNA precursors available in miRBase (release 22.1, October, 2018) [44] were mapped to the *B. tabaci* genome and compared to the miRDeep2 output in order to discriminate known and novel microRNA loci. Also, mature miRNAs available in the same database were aligned with the sequences predicted by miRDeep2 using the blastn algorithm. We considered as canonical miRNAs of OSW the reads with a perfect match or sharing up to two mismatches with the sequences in the database, regardless of their length. According to the miRBase guidelines on miRNA annotation [45,46] and with the concept of miRNA isoforms (isomiRs; sequences sharing very few mismatches with a miRNA and differing in length at the 5′ and/or 3′ ends) [47], the reads with more than two mismatches were considered as novel miRNAs in OSW. We propose to prepend the prefix ‘asp’ to the canonical miRNA name in order to associate it to OSW (e.g., asp-bantam, asp-miR-276a, asp-miR-317, etc.). IDs not present among the miRBase miRNAs were used for the novel miRNAs identified in this study, similarly preceded by the ‘asp’ prefix. Per each miRNA, the mature and star sequences were identified, as well as the indication of their 5p or 3p location in the precursor. Also, genomic coordinates of the precursors were determined in the *B. tabaci* genome. Normalized read count was expressed as reads per million (RPM) of reads mapping to the *B. tabaci* genome.

miRDeep2 calculates the number of raw reads per each predicted miRNA as the sum of the mature sequence and its isomiRs. In order to distinguish between read counts of mature miRNAs and isomiRs, we used the isomiRID tool [48]. Sequencing reads, mature and star sequences of miRNAs as well as their precursor sequences were submitted to isomiRID. Only isomiRs with up to one mismatch and differing in length up to ±2 and ±5 nt at the 5′ and 3′ ends, respectively, were retained. The output was manipulated in Microsoft Excel Professional 2016 for an arbitrary numbering of isomiRs. Also, the isomiR sequences were blasted against the miRBase database to find perfect matches with mature miRNAs. In order to assess the attitude of certain miRNA loci to generate more or less isomiRs, two indices were defined: the isomiR diversity index (IDI) was the number of different isomiRs relative to the normalized read count of a miRNA, and the isomiR abundance index (IAI) was the normalized read count of all isomiRs of a miRNA (excluding the mature sequence read count) relative to the normalized read count of the same miRNA.

## 3. Results and Discussion

### 3.1. *Aleurocanthus spiniferus* Was Found for the First Time on *Ailanthus altissima* in Italy

More than 100 puparia mounted on microscope slides were scrutinized. Based on taxonomic characters, the insects were identified as *A. spiniferus* because the number of marginal teeth ranged from 7 to 11, the crenulation sums 205–242 teeth, the ratio of longest spines were (1:9)–(1:17), the wide fringe represented 17.3%–30% of puparia width, cephalic eye spot were weakly defined, placed closer to the third sub-marginal spines rather than to the second ones, and microscopic papillae were situated between the sub-marginal spines [1,4]. Furthermore, the morphological identification was confirmed by a molecular study recently carried out on the same OSW population infesting the tree of heaven [3].

To the best of our knowledge, this is the first report of *A. spiniferus* infesting *A. altissima*. In fact, several studies have documented a number of pathogens and pests on *A. altissima*, but no mention of OSW has been reported [9,10,11,12,13]. This would suggest a host shift of OSW to *A. altissima*, but it cannot be excluded that the insect has infested this plant at extremely low population densities for a long time and it has not been observed or documented until now. Such a probable host shift merits a detailed investigation because it would add precious information about insect biology and ethology. It could be also studied whether insect endosymbionts, known as modulators of insect fitness [14,15], are involved in the colonization of this new plant host.

Moreover, the infestation by a so aggressive insect pest on the tree of heaven could have implications in the control of this alien and invasive plant. In our inspections, the tree of heaven was not particularly threatened by OSW, even under heavy infestation levels. Therefore, a possible contribution by OSW for the control of the tree of heaven cannot be hypothesized hitherto.

### 3.2. “*Candidatus* Portiera”, *Wolbachia*, and *Rickettsia* Were Identified in the OSW Microbiome

DGGE profiles of the 16S rDNA showed three different dominant bands. All samples shared a band that could be ascribed to the *Wolbachia* reference, a known endosymbiont of Aleyrodidae [49] and other insect species [50]. Another band occurring in all samples had no correspondence to any employed positive controls. Furthermore, four out of ten samples showed a band corresponding to the *Rickettsia* reference, another endosymbiont of several insect species [51].

Sequencing and BLAST analysis confirmed that the DNA sequences obtained from the excised bands matched *Rickettsia* spp. and *Wolbachia* spp. accessions, while those obtained from the band with no correspondence to the used references matched accessions of “*Candidatus* Portiera” Thao and Baumann (Gammaproteobacteria) (here referred to as “*Ca.* Portiera”).

**Figure 2:**
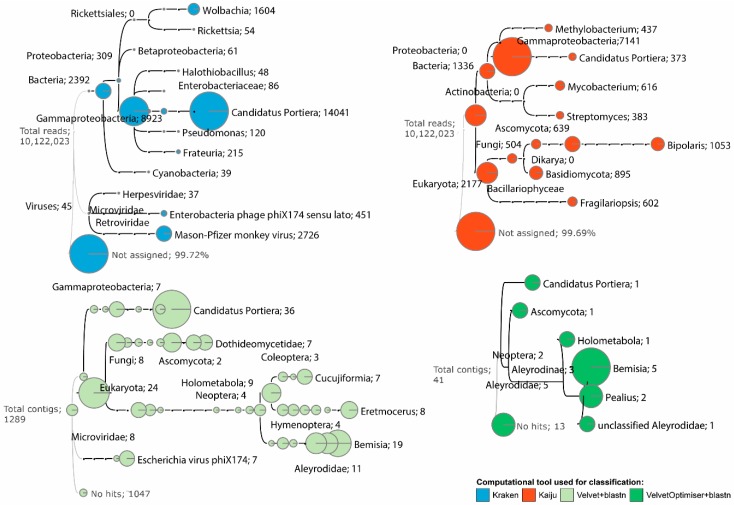
Dendrograms of the microbiome of *Aleurocanthus spiniferus* infesting *Ailanthus altissima* based on the results obtained using four different tools for taxonomic assignment of small RNA reads. The size of the circles is proportional to the number of reads or contigs placed in the taxon. Small RNA reads were subjected to Kraken and Kaiju or were assembled into contigs using Velvet or VelvetOptimiser prior to search sequence similarities against the BLAST nr database.

By using deep sequencing of sRNAs we were able to infer a more variegated microbiome composition (Figure 2). Most of the reads or contigs were unassigned to a Taxon. In fact, Kraken was able to classify only 0.28% reads, and Kaiju only 0.31%; among the 1289 contigs assembled with Velvet, only 242 were assigned to taxonomic units using a blastn search. On the other hand, 28 out of the 41 contigs assembled with VelvetOptimiser were classified by blastn.

Some microbial taxa were detected by different pipelines, while others only by one of them. Interestingly, the Aleyrodidae endosymbiont “*Ca.* Portiera” was identified by all the pipelines, and it was the most represented genus in three of them (except VelvetOptimiser). This bacterium is a well-known primary endosymbiont of Aleyrodidae species, including *Aleurocanthus woglumi* Ashby [49] and *B. tabaci* [14,52,53].

The Kraken tool detected considerable reads matching the *Wolbachia* genus. Also, the genera *Rickettsia*, *Pseudomonas*, *Frateuria,* and other taxa were identified by Kraken.

Kaiju identified the genera *Methylobacterium* Patt et al., *Mycobacterium* Lehmann and Neumann, and *Streptomyces* Waksman and Henrici among the bacteria, as well as Basidiomycota and Ascomycota (*Bipolaris* genus) among the fungi. Moreover, the genus *Fragillariopsis* (O’Meara) Hustedt (Bacillarophyceae) was also well represented. Besides *Rickettsia*, none of the latter microorganisms have been reported as Aleyrodidae endosymbionts. We did not find evidence for the presence of other secondary (facultative) endosymbionts of Aleyrodidae such as *Hamiltonella* Moran et al. [14,15,54,55] and another 14 genera [49]. Some species of the found guild of microorganisms might survive on the leaf surface, near or on the OWS; others seem frankly host-plant inhabitants that were taken by OSW during feeding and eventually concentrated in the filter chamber of the insect [56].

By using the contig-based pipelines (Velvet and VelvetOptimiser followed by blastn search), a number of sequences were assigned to Insecta, including *Bemisia* genus and other Aleyrodidae. No sequences were assigned to *Aleurocanthus* genus, probably because less represented than *Bemisia* or other Aleyrodidae in the databases. Remarkably, some contigs assembled by Velvet were assigned to the genus *Eretmocerus* Mercet (Hymenoptera), an Aleyrodidae parasitoid [57], and to the sub-order Cucujiformia (Coleoptera: Polyphaga), that encompasses plant-eating beetles and insect-predators [58].

Deep sequencing of insect sRNAs revealed that no plant-infecting viruses were present in the population of OSW hosted by the tree of heaven. When used with a virus database, Kraken detected three viral taxa: *Mason-Pfizer monkey virus* (M-PMV, Retroviridae; 2726 reads), Enterobacteriaceae phage phiX174 (Microviridae; 451 reads), and Herpesviridae (37 reads). Escherichia virus phiX174 was also detected by the Velvet + blastn pipeline (7 out of 242 contigs). Very likely, these hits were artifacts due to the forcing of search against viral sequences. Retroviridae (positive single-stranded RNA genome) and Herpesviridae (double-stranded DNA genome) infect vertebrates [59,60,61], but they have not been reported in insects, even after large screening surveys based on NGS [24,25,26,62]. Moreover, phiX174 (positive single-stranded DNA) is routinely used as a positive control in DNA sequencing on Illumina platforms [63].

### 3.3. Features of Small RNAs

**Figure 3:**
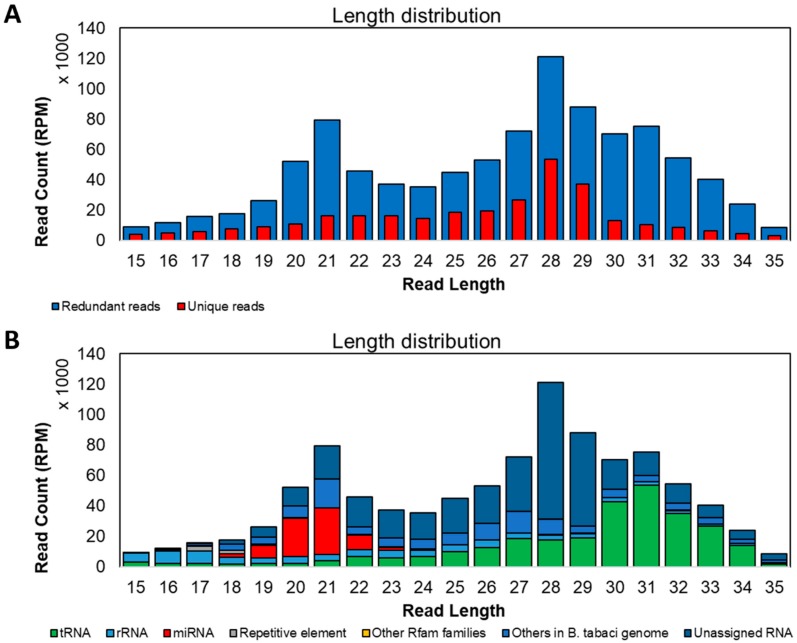
Length distribution of small RNAs of *Aleurocanthus spiniferus*: redundant, unique sequencing reads (**A**) and associated features (**B**).

**Figure 4:**
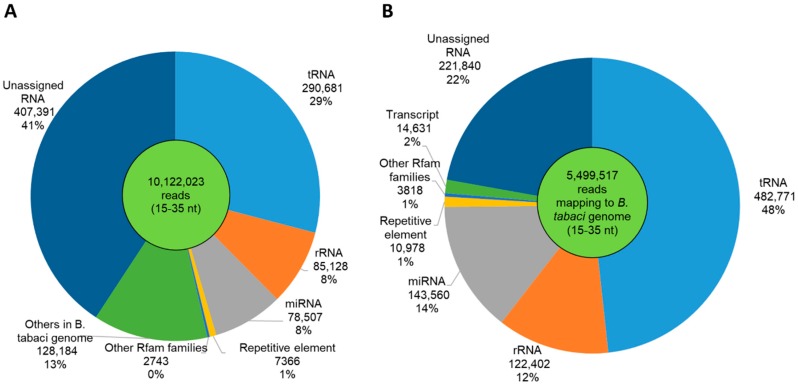
Features of small RNAs of *Aleurocanthus spiniferus* and their abundance in the library: sequencing reads of the entire library (**A**) and those mapping to *Bemisia tabaci* genome (**B**).

**Figure 5:**
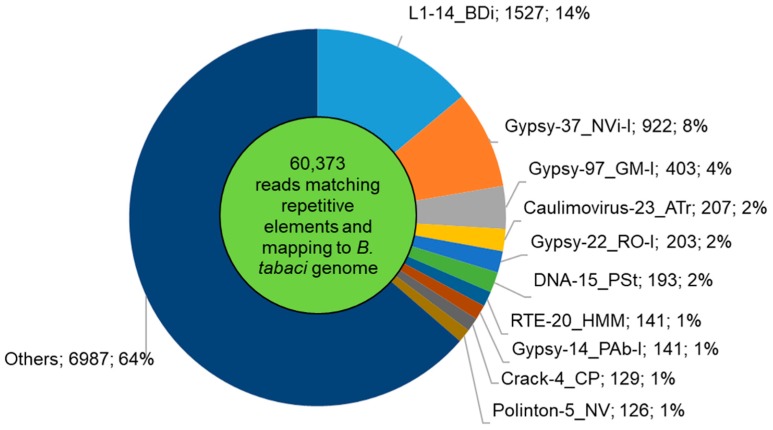
Abundance of several transposable element families in the *Aleurocanthus spiniferus* the library.

After quality filtering, we obtained 10,122,023 reads that were classified as tRNA, rRNA, miRNA, repetitive element, other Rfam families, others in *B. tabaci* genome, and unassigned RNA (Figure 3B). About 29% of reads were tRNAs, and 41% remained unassigned, while the remaining reads were classified as rRNAs, miRNAs, repetitive elements, other Rfam elements, or other features in the *B. tabaci* genome. About 54% of reads (5,499,517) were mapped to the *B. tabaci* genome. We retained only reads with length ranging from 15 to 35 nt, and their length distribution is shown in Figure 3. Frequency peaks were observed for sRNAs of 21 and 28 nt, in agreement with previous works on *B. tabaci* B and Q biotypes or other insects [64,65,66]. Thirty-six percent reads between 19 and 22 nt mapped to miRNAs, while 74% of 28 nt reads were unassigned (Figure 3B). They accounted for 8% of library (Figure 4A), and 12% of the reads matching the *B. tabaci* genome (Figure 3B; 5,499,517). Repetitive elements, accounting for 1% of library (Figure 4A), were assigned to reads of a wide range of length, but especially to the 17–18mers (Figure 3B). The families L1-14_BDi, Gypsy-37_NVi-I, Gypsy-97_GM-I, Caulimovirus-23_ATr, and Gypsy-22_RO-I were the top-five represented transposable elements (Figure 5).

### 3.4. MicroRNAs

Using the *B. tabaci* genome and miRBase database, the miRDeep2 tool identified 102 miRNAs in the OSW library. Sequence similarity analysis conducted with the blastn algorithm on these miRNAs against the miRBase database allowed us to classify the sequences into canonical (56) and novel miRNAs (46) (Table 1 and Table 2, respectively; also see Tables S1 and S2 for more details). Reads sharing up to two mismatches with the database sequences were considered as canonical OSW miRNAs and the rest as novel miRNAs. Differently, a threshold of four mismatches was used in two previous works on *B. tabaci*, where more miRNAs than in our research were identified [64,65,67].

Canonical miRNAs were identified in 41 out of 19,751 scaffolds of the *B. tabaci* genome. MicroRNA precursors were predicted by miRDeep2, and 21 of them were highly similar to those available in miRBase (e.g., dme-mir-276a, dqu-mir-317, dme-mir-184, etc.). Several miRNAs were predicted to originate from diverse precursors, either with different nt sequences or with different positions in the genome. However, we cannot conclude if all these miRNAs actually have different precursors in OSW because we used the *B. tabaci* genome, and even the complete assembly of this genome into chromosomes is still pending. We found that asp-miR-iab-4 and asp-miR-iab-8 originated from the minus and plus DNA strand, respectively, of the same miRNA locus (Table 1), as already reported in *D. melanogaster* [68,69]. A similar scenario occurred for asp-miR-134420, which originated the same miRNA/miRNA* from both DNA strands. For most canonical miRNAs, we detected both mature and star sequences (distinguished based on their counts), supporting the reliability of those miRNAs in OSW according to the miRBase guidelines [45,46].

Among the 46 novel miRNAs detected by miRDeep2, at least four miRNAs (asp-miR-111690, asp-miR-131950, asp-miR-73390, and asp-miR-131930p; Table 2) had a high score (>10), significant randfold *p*-value, high abundance, and/or an expressed star sequence, indicating their high confidence. Moreover, miRNAs such as asp-miR-131930-5p and asp-miR-129380-3p had isomiRs matching perfectly dme-miR-12-5p and dme-miR-981-3p, respectively (data not shown), that were also found as mature miRNAs in previous works [64,65]. Very likely, the remaining novel miRNAs detected by miRDeep2 should be considered with low confidence, and they would need further confirmation.

As expected, most known OSW miRNAs were orthologous of miRNAs previously described in other arthropods (Table S1). In fact, by analyzing miRNAs of three hemimetabolan (*Blattella germanica* L., *Locusta migratoria* L., and *Acyrthosiphon pisum* Harris) and four holometabolan species [*Apis mellifera* L., *Tribolium castaneum* (Herbst), *Bombyx mori* L., and *D. melanogaster* Meigen], Ylla and coworkers [70] concluded that insects share a conserved microRNA toolkit of 65 families exhibiting very low variation. The asp-bantam-3p, asp-miR-276a-3p, asp-miR-317-3p, asp-miR-750-3p, and asp-mir-8-3p were the top-five miRNAs for abundance in the sampled OSW (3–116 thousand RPM for the mature sequence and its isomiRs), and asp-miR-3049-3p, asp-miR-2796-3p, asp-miR-965-3p, asp-miR-iab-4-5p, and asp-miR-iab-8-3p the less represented (0.4–16 RPM for the mature sequence and its isomiRs). The abundance of these miRNAs was in agreement with miRBase (that reports, for example, 5 × 10^4^ RPM of dme-bantam as an average of 49 experiments; we observed 1 × 10^3^ RPM) and with previous observations in *B. tabaci* [64,65], confirming their conserved (though multiple) functions in related species.

MicroRNA bantam promotes cell proliferation, inhibits apoptosis, maintains germline stem cells, limits scaling growth of dendrites, and regulates circadian rhythm clock [66,71,72]. MicroRNA miR-8 prevents neurodegeneration in the brain, is involved in the development of wings and eyes, promotes organismal growth in larvae, and controls the morphology of neuromuscular junctions [71]. MicroRNA miR-317 is critical for *Drosophila* survival, as it is a negative regulator of *Drosophila* Toll signaling immune response [73]. MicroRNA miR-750 is a cluster conserved in several hemimetabolan and holometabolan insects but lost in *D. melanogaster* [70]. It was the most abundant miRNA in silkworm infected by *Bombyx mori* *cytoplasmic polyhedrosis virus* (BmCPV) [67]. MicroRNA miR-276-5p fine-tunes the duration of the reproductive cycle in *Anopheles* [74] and promotes egg-hatching synchrony in *L. migratoria* [75]. Overall low expression of miRNAs, however, does not mean that they are of minor importance in the metabolism. MicroRNA mir-iab-4, for example, causes a dominant homeotic transformation of halteres to wings in *D. melanogaster* [76]. We found only 4.7 RPM of asp-miR-iab-4-5p, and miRBase reports 438 RPM as an average of 45 experiments for dme-miR-iab-4-5p. The abundance of some OSW miRNAs deviated from the general trend occurring in other insects. For example, let-7 had an intermediate abundance in OSW (745.2 RPM for the mature sequence) but was highly expressed in *B. tabaci* (1–2 × 10^5^) and induced by TYLCCNV infection [64,65]. MicroRNA let-7-5p, along with miR-100-5p and miR-125-5p, emerged as an important factor in the metamorphic stage of *B. germanica* [77].

The tool miRDeep2 counts both the mature sequence and its isomiRs for each detected miRNA and miRNA star. In order to count separately the mature sequences and the isomiRs, we used the tool isomiRID and then extracted the counts from its output. Due to their abundance, isomiRs appeared particularly important to assess the expression level of a miRNA. For example, asp-bantam, which was the most abundant miRNA in the OSW library, accounted for 115,692.2 RPM (isomiRID count, Table S4), resulting from 33,695 RPM of mature miRNA and 79,930.3 RPM of isomiRs (606 different sequences). The isomiR diversity index (IDI), here defined as the number of different isomiR sequences relative to the mature miRNA count, decreased with the expression level of miRNAs, meaning that highly abundant miRNAs had a smaller number of different isomiRs (Figure S1). However, such a correlation was not strong (*R*^2^ = 0.42), so that miRNAs like asp-miR-2a-3p had 281.9 RPM of mature miRNAs and 181 different isomiRs (IDI = 0.64), while miRNAs like asp-miR-87a-3p had 240.57 RPM of mature miRNA and 31 isomiR sequences (IDI = 0.13). The isomiR abundance index (IAI), here defined as the total isomiR count relative to the mature miRNA count, did not depend on the miRNA abundance. In fact, miRNAs with similar abundance such as the two above-mentioned miRNAs asp-miR-2a-3p and asp-miR-87a-3p had a different IAI (1249.29 and 114.16 RPM of isomiRs, respectively, corresponding to IDIs of 4.43 and 0.47). In contrast, miRNAs with largely different expression levels such as asp-miR-317-3p (811.34 RPM of mature miRNA) and asp-miR-305-5p (99.1 RPM) had similar IDIs, i.e., 3.81 and 3.67, respectively.

By analyzing the isomiRs, we observed that A to C substitution was the most abundant occurrence (data not shown), which is not in line with Naqvi et al. [78], who found that most of the substitutions comprised A to G and C to T events.

## 4. Conclusions

We report the first documented stable infestation of *A. altissima* by OSW in Italy, which very likely originated from the neighboring citrus trees, the main host plants of OSW. A possible contribution by OSW for the control of the tree of heaven cannot be hypothesized hitherto because, in our observations, the plant tolerated well the OSW infestation.

By using DGGE and sRNA deep sequencing, we provide sound evidence on the presence of the primary endosymbiont “*Ca.* Portiera” and other endosymbionts such as *Wolbachia* spp. and *Rickettsia* spp. For the first time, we used sRNA-Seq to profile an insect microbiome and paved the way to compare this technique with the most widely used metagenomics approaches. Moreover, further investigations are needed to assess whether endosymbionts play a role in the interaction between OSW and the tree of heaven and whether the OSW microbiome can be harnessed to develop new management strategies against either the OSW or the tree of heaven.

Finally, we identified 56 canonical and 46 novel miRNAs in OSW, the latter group including at least four miRNAs detected with a high confidence level.

## Figures and Tables

**Table 1:** Canonical miRNAs detected by miRDeep2 tool using *Aleurocanthus spiniferus* small RNA reads, *Bemisia tabaci* genome, and miRBase database.

Provisional MiRNA Name (Mature/Star)	miRDeep2 ID	miRDeep2 Score	Read Count (RPM)		Example of miRBase miRNA with the Same Seed	Alignment between *A. spiniferus* miRNA (up) and miRbase miRNA (Bottom)	Precursor Coordinate in the *Bemisia tabaci* Genome
			Mature	Star			
asp-bantam-3p asp-bantam-5p	NW_017547202.1_5067	5.2	115,812.7	5.5	dme-bantam-3p	UGAGAUCAUCGUGAAAGCUG .........UU.........AUU	NW_017547202.1:1932321..1932378: +
asp-miR-276a-3p asp-miR-276a-5p	NW_017547107.1_1308	5 × 10^4^	17,936.8	10.8	dme-miR-276a-3p	UAGGAACUUCAUACCGUGCUC .....................U	NW_017547107.1:5694695..5694839: − ^b^
asp-miR-317-3p asp-miR-317-5p	NW_017550575.1_11879	1.1 × 10^4^	4034.0	4.0	dqu-miR-317-3p	UGAACACAGCUGGUGGUAUCUCAG ........................	NW_017550575.1:773861..774000: + ^b^
asp-miR-750-3p asp-miR-750-5p	NW_017547443.1_8884	9.4 × 10^3^	3348.7	18.5	ame-miR-750-3p	CCAGAUCUAACUCUUCCAGCUC ......................	NW_017547443.1:370244..370308: −
asp-mir-8-3p asp-mir-8-5p	NW_017547115.1_2104	4.4	3101.0	3.7	api-mir-8	UAAUACUGUCAGGUAAUGAU ....................GUC	NW_017547115.1:1766399..1766456: −
asp-miR-184-3p asp-miR-184-5p	NW_017563531.1_15559	5.1	26.2	0	dme-miR-184-3p	UGGACGGAAAACUGAUAAGGGU ........G............C	NW_017563531.1:2205..2265: +
	NW_017552810.1_13306	4.8	26.2	0		UGGACGGAAAACUGAUAAGGGU ........G............C	NW_017552810.1:1753402..1753462: −
	NW_017547113.1_1681	5	3074.1	0.6		UGGACGGAGAACUGAUAAGGG .....................C	NW_017547113.1:1014029..1014184: + ^b^
asp-miR-2765-5p asp-miR-2765-3p	NW_017547098.1_412	3.8 × 10^3^	1355.4	5.1	ame-miR-2765-5p	UGGUAACUCCACCACCGUUGG .....................C	NW_017547098.1:2423456..2423512: +
	NW_017547098.1_410	4.8	2424.9	0		UGGUAACUCCACACCACCGUUGG ............--.........C	NW_017547098.1:2422100..2422160: +
asp-miR-100-5p asp-miR-100-3p	NW_017547299.1_7860	4.9 × 10^3^	1758.5	2.0	dme-miR-100-5p	AACCCGUAGAUCCGAACUUGU .....................G	NW_017547299.1:803546..803601: +
asp-miR-13a-3p asp-miR-13a-5p	NW_017547352.1_8563	4.6 × 10^3^	1617.4	49.2	tca-miR-13a-3p	UAUCACAGCCACUUUGAUGAAC ....................G.	NW_017547352.1:37273..37334: +
asp-miR-2a-3p asp-miR-2a-5p	NW_017547352.1_8561	2.9 × 10^3^	1039.5	0.2	mse-miR-2a	UCACAGCCAGCUUUGAUGAGCA ......................	NW_017547352.1:36468..36531: +
	NW_017547352.1_8569	1.7 × 10^3^	636.6	0.9	dme-miR-2a-3p	UAUCACAGCCAGCUUUGAUGA .....................GC	NW_017547352.1:40230..40282: +
	NW_017547352.1_8567	5.4	638.8	1.1			NW_017547352.1:40048..40201: + ^b^
	NW_017561640.1_15257	1.7 × 10^3^	636.6	0.9			NW_017561640.1:532..683: + *^b^*
	NW_017561640.1_15255	5.4	638.8	1.1			NW_017561640.1:382..438: +
asp-miR-315-5p asp-miR-315-3p	NW_017547334.1_8451	4.8	1501.8	0	dme-miR-315-5p	UUUUGAUUGUUGCUCAGAAAGC ......................	NW_017547334.1:1004963..1005023: −
asp-miR-9a-5p asp-miR-9a-3p	NW_017551177.1_12283	3.9 × 10^3^	1395.9	22.2	dme-miR-9a-5p	UCUUUGGUUACCUAGCUGUAUG ..........T...........	NW_017551177.1:699591..699652: −
asp-miR-277-3p asp-miR-277-5p	NW_017550575.1_11881	2.9 × 10^3^	1062.8	2.0	dme-miR-277-3p	UAAAUGCACUAUCUGGUACGAC ......................A	NW_017550575.1:818061..818121: +
asp-miR-71-3p asp-miR-71-5p	NW_017547352.1_8559	2.8 × 10^3^	860.6	139.8	tca-miR-71-3p	UCUCACUACCUUGUCUUUCAU .....................G	NW_017547352.1:36323..36463: + ^b^
asp-miR-14-3p asp-miR-14-5p	NW_017547098.1_493	2.5 × 10^3^	847.3	57.8	dme-miR-14-3p	UCAGUCUUUUUCUCUCUCCUA .....................U	NW_017547098.1:8908667..8908729: +
asp-miR-275-3p asp-miR-275-5p	NW_017547297.1_7629	5.6	820.1	101.1	dme-miR-275-3p	UCAGGUACCUGAAGUAGCGCG .....................CG	NW_017547297.1:1465090..1465268: + ^b^
asp-let-7-5p asp-let-7-3p	NW_017547299.1_7862	4.8	745.2	6.2	dme-let-7-5p	UGAGGUAGUAGGUUGUAUAG ....................U	NW_017547299.1:803734..803802: +
asp-miR-263a-5p asp-miR-263a-3p	NW_017547161.1_3192	5.1	696.1	4.8	dme-miR-263a-5p	AAUGGCACUAGAAGAAUUCACG .........G............GG	NW_017547161.1:912282..912340: +
asp-miR-31-5p asp-miR-31-5p	NW_017547107.1_1345	5	654.4	0	aca-miR-31-5p	AGGCAAGAUGUUGGCAUAGC ....................U	NW_017547107.1:9048192..9048254: −
asp-miR-306-5p asp-miR-306-3p	NW_017547320.1_8138	5.3	597.3	1.5	dme-miR-306-5p	UCAGGUACUGAGUGACUCU .........G.........	NW_017547320.1:2159894..2159951: +
asp-miR-305-5p asp-miR-305-3p	NW_017547297.1_7632	5.1	522.0	2.4	dme-miR-305-5p	AUUGUACUUCAUCAGGUGCUC .....................UG	NW_017547297.1:1466885..1466944: +
asp-miR-92b-3p asp-miR-92b-5p	NW_017564582.1_15992	0.6 *^a^*	488.4	0	dme-miR-92b-3p	AAUUGCACUAGUCCCGGCCUG .....................C	NW_017564582.1:513918..513972: −
asp-miR-13b-3p asp-miR-13b-5p	NW_017561640.1_15253	1.1 × 10^3^	311.8	109.2	bmo-miR-13b	UAUCACAGCCAUUUUUGACGUGCC ....................A.U	NW_017561640.1:204..265:+
	NW_017547352.1_8565						NW_017547352.1:39883..39944: +
asp-miR-34-5p asp-miR-34-3p	NW_017550575.1_11884	5.1	297.1	15.4	dme-miR-34-5p	UGGCAGUGUGGUUAGCUGGUUG ......................UG	NW_017550575.1:854739..854841: + ^b^
asp-miR-87a-3p asp-miR-87a-5p	NW_017547298.1_7717	2.8 × 10^2^	98.7	2.8	tca-miR-87a-3p	GUGAGCAAAGUUUCAGGUGUG .....................	NW_017547298.1:2017127..2017227: +
	NW_017547298.1_7719	8.2 × 10^2^	285.8	5.7	tca-miR-87a-3p	GUGAGCAACGUAUCAGGUGUC ........A..U........GA	NW_017547298.1:2017271..2017332: +
asp-miR-993-3p asp-miR-993-5p	NW_017547290.1_7299	9.5 × 10^2^	276.9	64.2	dme-miR-993-3p	GAAGCUCGUCUCUACAGGUAUC ......................U	NW_017547290.1:727642..727815: + ^b^
asp-miR-125-5p asp-miR-125-3p	NW_017547299.1_7864	7.8 × 10^2^	262.4	14.9	dme-miR-125-5p	UCCCUGAGACCCUAAUUUGUG ...............C.....A	NW_017547299.1:822960..823021: +
asp-miR-927-3p asp-miR-927-5p	NW_017547282.1_7013	8.7 × 10^2^	227.3	83.3	dme-miR-927-3p	CAAAGCGUUUGAAUUCUGGAA ...........G......A..C	NW_017547282.1:1940947..1941008: +
asp-miR-281-2-5p asp-miR-281-2-3p	NW_017561619.1_15248	8.1 × 10^2^	219.8	68.8	dme-miR-281-2-5p	AAGAGAGCUAUCCUUCGACAG .............G.......U	NW_017561619.1:12115..12174: +
asp-miR-252a-5p asp-miR-252a-3p	NW_017554870.1_13884	5.9 × 10^2a^	212.4	0.2	dpu-miR-252a	CUAAGUACUCCGUGCCGCAGG ..........-..........AG	NW_017554870.1:193643..193702: +
asp-miR-1-3p asp-miR-1-5p	NW_017547904.1_9568	3.9 × 10^2^	137.8	2.2	dme-miR-1-3p	UGGAAUGUAAAGAAGUAUGGA .....................G	NW_017547904.1:1209882..1209940: −
asp-miR-190-5p asp-miR-190-3p	NW_017547096.1_359	3.3 × 10^2^	117.3	0.6	dme-miR-190-5p	AGAUAUGUUUGAUAUUCUUGGUU .......................G	NW_017547096.1:1449782..1449864: − ^b^
asp-miR-279b-3p asp-miR-279b-5p	NW_017547145.1_3172	5.2	114.4	0	pxy-miR-279b-3p	UGACUAGAUUUUCACUCAUUC ...................C.	NW_017547145.1:2285467..2285523: −
asp-miR-92a-3p asp-miR-92a-5p	NW_017564582.1_15994	5.1	86.9	0	dme-miR-92a-3p	UAUUGCACUUGUCCCGGCC C..................UAU	NW_017564582.1:514087..514138: −
asp-miR-133-3p asp-miR-133-5p	NW_017547904.1_9564	2.3 × 10^2^	83.8	0.2	dme-miR-133-3p	UUGGUCCCCUUCAACCAGCUG .....................U	NW_017547904.1:1012752..1012934: − ^b^
asp-miR-263b-5p asp-miR-263b-3p	NW_017547161.1_3196	2.4 × 10^2^	81.3	3.7	api-miR-263b	CUUGGCACUGGAAGAAUUCACAG .......................A	NW_017547161.1:950587..950647: +
asp-miR-252b-5p asp-miR-252b-3p	NW_017554870.1_13882	2 × 10 ^2a^	72.9	0.9	api-miR-252b	CUAAGUAGUAGCGCCGAAGGUG ...............A.C....A	NW_017554870.1:149158..149217: +
asp-miR-124-3p asp-miR-124-5p	NW_017557672.1_14877	0.3 ^a^	64.0	37.4	dme-miR-124-3p	UAAGGCACGCGGUGAAUGCC ....................AAG	NW_017557672.1:97524..97666: + ^b^
asp-miR-375-3p asp-miR-375-5p	NW_017547090.1_76	1.5 × 10^2^	53.5	1.5	ame-miR-375-3p	UUUGUUCGUUCGGCUCGAGUU .....................A	NW_017547090.1:1647645..1647829: − ^b^
asp-miR-10-5p asp-miR-10-3p	NW_017547290.1_7361	4.4	51.8	0.9	oar-miR-10a	UACCCUGUAGAUCCGAAAUU .................U..G	NW_017547290.1:727642..727815: − ^b^
asp-miR-996-3p asp-miR-996-5p	NW_017553907.1_13657	1.5 × 10^2^	46.2	9.2	api-miR-996	UGACUAGAGUUACACUCGUC ....................A	NW_017553907.1:824153..824211: −
asp-miR-7-5p asp-miR-7-3p	NW_017556795.1_14461	1.1 × 10^2^	38.2	1.1	dme-miR-7-5p	UGGAAGACUAGUGAUUUUGUUGU .......................	NW_017556795.1:161510..161612: − ^b^
asp-miR-210-3p asp-miR-210-5p	NW_017550873.1_12017	4.8	36.5	0.4	dme-miR-210-3p	CUUGUGCGUGUGACAGCGGC ....................UAU	NW_017550873.1:911604..911762: + ^b^
asp-miR-1000-5p asp-miR-1000-3p	NW_017547246.1_6234	5	32.0	0.9	dme-miR-1000-5p	AUAUUGUCCUGUCACAGCAG ....................U	NW_017547246.1:2709831..2709911: + ^b^
asp-miR-929-5p asp-miR-929-3p	NW_017547330.1_8238	5	25.6	0.2	dme-miR-929-5p	AAAUUGACUCUAGUAGGGAG ....................UC	NW_017547330.1:415939..415997: +
asp-miR-137-3p asp-miR-137-5p	NW_017547181.1_4201	73	25.1	0.4	dme-miR-137-3p	UAUUGCUUGAGAAUACACGUA .....................G	NW_017547181.1:4906197..4906365: + ^b^
asp-miR-29b-3p asp-miR-29b-5p	NW_017547261.1_6573	4.3	23.6	0	aca-miR-29b	UAGCACCAUUUGAAAUCAGUG .....................	NW_017547261.1:4373495..4373551: +
	NW_017548020.1_10048						NW_017548020.1:5969402..5969450: −
asp-miR-971b-5p asp-miR-971b-3p	NW_017547177.1_3772	72	21.6	2.8	tca-miR-971b-5p	CACUCUAAGCUCGAACAUCAAG .........U.U..........C	NW_017547177.1:2847368..2847428: +
asp-miR-307a-3p asp-miR-307a-5p	NW_017547096.1_364	4.8	20.7	0	dme-miR-307a-3p	UCACAACCUCCUUGAGUGAG ....................CGA	NW_017547096.1:1789984..1790139: − ^b^
asp-miR-3049-3p asp-miR-3049-5p	NW_017547142.1_3035	69	16.2	7.0	ame-miR-3049-3p	UCCGUCCAACUCUUUUCCGCC ............C......U.U	NW_017547142.1:3651122..3651182: +
asp-miR-2796-3p asp-miR-2796-5p	NW_017547276.1_6782	50	15.8	0.7	ame-miR-2796-3p	GUAGGCCGGCGGAAACUACUUG ......................C	NW_017547276.1:164674..164899: − ^b^
asp-miR-998-3p asp-miR-998-5p	NW_017547112.1_1536	5.3	14.5	0	api-miR-998	UAGCACCAUGGAAUUCAGCUU ....................G	NW_017547112.1:1633093..1633150: −
asp-miR-1175-3p asp-miR-1175-5p	NW_017547443.1_8882	3.3	13.5	0	dme-miR-958-3p	UGAGAUUCAACUCCUCCAUC ....................	NW_017547443.1:363797..363852: −
asp-miR-965-3p asp-miR-965-5p	NW_017551483.1_12566	4.4	10.7	3.7	dme-miR-965-3p	UAAGCGUAUAGCUUUUCCCC ....................UU	NW_017551483.1:4663002..4663057: −
asp-miR-iab-4-5p asp-miR-iab-4-3p	NW_017549609.1_11445	4.7	4.7	0.7	dme-miR-iab-4-5p	ACGUAUACUAAAUGUAUCCU .........G..........GA	NW_017549609.1:2987912..2987972: −
asp-miR-iab-8-3p asp-miR-iab-8-5p	NW_017549609.1_11368	1.5	0.4	0.6	dps-miR-iab-8-3p	AGGAUACAUUUAGUAUACGUC ..........C.........AUA	NW_017549609.1:2987914..2987973: +

^a^ Non-significant randfold *p*-value. ^b^ Significant (e-value < 0.01) BLAST alignment between miRBase precursor and *Bemisia tabaci* genome.

**Table 2:** Novel miRNAs (more than two mismatches shared with miRBase sequences) detected by miRDeep2 tool using *Aleurocanthus spiniferus* small RNA reads, *Bemisia tabaci* genome, and miRBase 22 database.

Provisional miRNA Name (Mature/Star)	miRDeep2 ID	miRDeep2 Score	Read Count (RPM)		Example of miRBase miRNA with the Same Seed	Alignment between *A. spiniferus* miRNA (up) and miRbase miRNA (Bottom)	Precursor Coordinate in the *Bemisia tabaci* Genome
			Mature	Star			
asp-miR-111690-5p asp-miR-111690-3p	NW_017549178.1_11169	2.2 × 10^2^	68.9	9.0	efu-mir-9235b	UGUGAUGUGCCUGUGGGCUUU UAAU.U.......G.......AAG	NW_017549178.1:3495258..3495320: −
asp-miR-131950-5p asp-miR-131950-3p	NW_017552654.1_13195	1 × 10^2^	29.6	6.8	dme-miR-304-5p	UAAUCUCAUACAGUUAUCCAUGA ..........UUUG..AAUG...G	NW_017552654.1:189212..189276: −
asp-miR-73390-3p asp-miR-73390-5p	NW_017547290.1_7339	11	2.0	0.6	rno-miR-3590-3p	UGUCACAUUCGAAAAGAGCUG UA.CACA..GU..........C	NW_017547290.1:2474877..2474936: +
asp-miR-131930-5p asp-miR-131930-3p	NW_017552654.1_13193	4.5	1467.2	22.2	dme-miR-12-5p	UGAGUAUUACAUCAGAUAUUU ...............G..C.GGU	NW_017552654.1:186778..186837: −
asp-miR-81360-5p asp-miR-81360-3p	NW_017547320.1_8136	4.3	405.1	0	dme-miR-9a-5p	UCUUUGGUAUUCUAGCUGUAG ........UA..........UGA	NW_017547320.1:2154975..2155034: +
asp-miR-36450-5p asp-miR-36450-3p	NW_017547174.1_3645	5.2	343.1	0	dme-miR-5-5p	UAAGGAACUAGGAAUGAGAUG A.......G.UCGU..U...AUG	NW_017547174.1:1937885..1937946: −
asp-miR-129380-3p asp-miR-129380-5p	NW_017552322.1_12938	5	65.8	0	dme-miR-981-3p	UUCGUUGUCGCCGAAAACUCGUC ..........A.....C..GCA	NW_017552322.1:646402..646467: −
asp-miR-38410-3p asp-miR-38410-5p	NW_017547177.1_3841	2.8	46.9	0	zma-miR396e-3p	ACUUUGGCCGUGAAAGCCGUG ..U.AA..........GGAAG	NW_017547177.1:5603872..5603954: +
asp-miR-161110-3p asp-miR-161110-5p	NW_017564655.1_16111	5.2	36.5	0	api-miR-3016	UUUGGUAAAAGAUAGCCGGUA A.......C.C.....UCU.UAG	NW_017564655.1:2288547..2288606: +
asp-miR-1011900-3p asp-miR-1011900-5p	NW_017548094.1_10119	5.1	33.3	0	dme-miR-993-3p	CAAGCUCGUUGAAGUAUACCC G........CUCUAC.GGUAUCU	NW_017548094.1:36089..36137: +
asp-miR-64150-3p asp-miR-64150-5p	NW_017547257.1_6415	1.3 ^a^	26.7	0	bmo-miR-3322	CCUCGUCGGCGUCGGCGGC U........UU.GAAGUUGGCUAG	NW_017547257.1:1451280..1451335: +
asp-miR-22650-3p asp-miR-22650-5p	NW_017547116.1_2265	0.4 ^a^	11.8	0	ame-miR-9883-5p	GUCGGGCGUAGUUAGUACU U.......GGC.CG.GCGAGA	NW_017547116.1:7925742..7925790: +
	NW_017548020.1_10048		0	0			NW_017548020.1:5969402..5969450: −
	NW_017547090.1_19	0 ^a^	21.8	0		GUCGGGCGUAGUUAGUACUUAGAU U.......GGC.CG.GCGAGA	NW_017547090.1:1477845..1477898: +
asp-miR-33410-5p asp-miR-33410-3p	NW_017547161.1_3341	3.2	18.9	0	mmu-miR-6976-5p	CAGGGAAUUUUUGACCAAAA .......G..GA..GG....UUG	NW_017547161.1:1252883..1252946: −
asp-miR-161130-3p asp-miR-161130-5p	NW_017564655.1_16113	5	13.5	0	dme-miR-9c-3p	ACUUUGGUAAAAACAGCUGUG UAAAG....UA..CC..AGCUC	NW_017564655.1:2292773..2292828: +
asp-miR-144320-5p asp-miR-144320-3p	NW_017556720.1_14432	5.8	12.5	0	eca-miR-9128	ACUCGCGCGGGCACCGUCGU CUGGUG......U....CA....AC	NW_017556720.1:451897..451955: +
	NW_017549581.1_11314		0	0			NW_017549581.1:39602..39660: +
	NW_017555149.1_13938		0	0			NW_017555149.1:1072638..1072696: +
asp-miR-169840-3p asp-miR-169840-5p	NW_017566817.1_16984	3.6	7.5	0	eca-miR-9047	AGUUCCCGGGUUUCGGCACC U........C.GA.CCUGG.UAGG	NW_017566817.1:2966919..2966963: −
asp-miR-81390-3p asp-miR-81390-5p	NW_017547320.1_8139	4.7	5.3	0	dme-miR-4-3p	AUAAAGCUGGAUUACCAAAGC ........A..CA....UU.A	NW_017547320.1:2163341..2163400: +
asp-miR-147030-3p asp-miR-147030-5p	NW_017557621.1_14703	0.8 ^a^	5.3	0	mmu-miR-7648-3p	CGGCGCGGCCCGGGCGGCGG A...UG........AC....G	NW_017557621.1:10736677..10736742: +
asp-miR-1850-5p asp-miR-1850-3p	NW_017547093.1_185	1.4 ^a^	4.7	0	hbv-miR-B4	UCGGGGCGGCCGUUGCCG A.......UGGU.G.UG.GCG	NW_017547093.1:1632977..1633017: −
asp-miR-129190-3p asp-miR-129190-5p	NW_017552322.1_12919	3.8	4.4	0	mdv1-miR-M10-3p	UCCCGGAGAAAUUUCGAGCU ..U..U...G.......UAACA	NW_017552322.1:437934..437995: +
asp-miR-29250-5p asp-miR-29250-3p	NW_017547136.1_2925	0 ^a^	4.4	0	mdo-miR-7380-5p	AGGUCCCAGGUUCGAUCCCU .........C.CUAC...UCAU	NW_017547136.1:599807..599880: −
	NW_017547136.1_2927	0 ^a^	0	0		AGGUCCCAGGUUCGAUCCCU ...........CUAC...UCAU	NW_017547136.1:603764..603837: −
asp-miR-75080-3p asp-miR-75080-5p	NW_017547292.1_7508	0.4 ^a^	4.0	0	gga-miR-6592-3p	CGCGUCUCCUCCCUCGGA A........CG...GACGGCC	NW_017547292.1:2958777..2958832: −
asp-miR-87590-3p asp-miR-87590-5p	NW_017547427.1_8759	5	3.8	0	atr-miR8572	CGACCCGGUCGGCGUCGG U..........UU.GAAUCCUCUC	NW_017547427.1:2779263..2779325: +
asp-miR-140930-5p asp-miR-140930-3p	NW_017555629.1_14093	0 ^a^	3.6	0	dme-miR-959-5p	UUAGUACUUAGAUGGGAGAC U.......CG.G.U.AUA.AG	NW_017555629.1:127516..127600: −
asp-miR-151900-3p asp-miR-151900-5p	NW_017560566.1_15190	0.8	3.3	0	bta-miR-11982	AUUACCCGGCGCCUCCACC UU.............CUGCGGGU	NW_017560566.1:657..718: −
asp-miR-8520-5p asp-miR-8520-3p	NW_017547103.1_852	0.8 ^a^	3.1	0	mmu-miR-3081-5p	GGCCGCGAGCGUGGAGUGGUGA .A.UG....U.....C.....	NW_017547103.1:943997..944071: +
asp-miR-35640-5p asp-miR-35640-3p	NW_017547174.1_3564	5	2.9	0	dme-miR-5-5p	UAAGGAACUGUUUGAUGUGGU A.......GA.CGUUGUGAUAUG	NW_017547174.1:1945099..1945157: +
asp-miR-70610-3p asp-miR-70610-5p	NW_017547282.1_7061	3.3	2.9	0	ame-miR-750-3p	CAGCUCUAACUCUUAAAGCUG .CAGA..........CCAG.C	NW_017547282.1:4449832..4449878: +
asp-miR-29460-5p asp-miR-29460-3p	NW_017547139.1_2946	1.2	2.9	0	ame-bantam-3p	AGAUCCUUGUGACAGCUU UG.....A......A....GAUU	NW_017547139.1:273731..273785: +
asp-miR-46090-5p asp-miR-46090-3p	NW_017547184.1_4609	4.2	2.4	0	dvi-miR-9709-5p	UUGCACUUCGGUAUCUCCAACCUAU ..........C.U.U.UAU..AACG	NW_017547184.1:3061811..3061874: −
asp-miR-109-5p asp-miR-42860-3p	NW_017547182.1_4286	3.9	2.4	0	gsa-miR-10b-5p	UGUAGACCUGAAUCA AACCC........C....UUGA	NW_017547182.1:1044722..1044786: +
asp-miR-49480-3p asp-miR-49480-5p	NW_017547098.1_445	1.9 ^a^	2.4	0	dme-miR-4948-5p	CGGCGGGCCGGGCGGCGU ........GU....UGCGUGAU	NW_017547098.1:4979831..4979950: +
asp-miR-125030-5p asp-miR-125030-3p	NW_017551483.1_12503	0.9	2.2	0	efu-miR-9212	UUCGCAUGGAGUCAUCUGUUAU ACA........GG.....GAGA	NW_017551483.1:448169..448229: −
asp-miR-165650-5p asp-miR-165650-3p	NW_017566689.1_16565	2.9	2.0	0	hsa-miR-10523-5p	GACAAUGAUGACAAAAUUUGGU ...........G..G.CC..AGGA	NW_017566689.1:494135..494184: −
asp-miR-54140-5p asp-miR-54140-3p	NW_017547211.1_5414	4.6	1.8	0	hsa-miR-1538	GGGCCCGGGGUUCGAUUCC C........C.G.UGC.GUUCC	NW_017547211.1:1082752..1082825: +
asp-miR-1077800-3p asp-miR-1077800-5p	NW_017549038.1_10778	4.4	1.5	0.4	pxy-miR-8536b-3p	AGGUAUCUGAGCGAUGUCCCCAC CC.....C...U.....A.U.	NW_017549038.1:8023737..8023796: +
	NW_017551711.1_12713		0	0			NW_017551711.1:428822..428883: −
asp-miR-131970-5p asp-miR-131970-3p	NW_017552654.1_13197	1.7	1.3	0	dme-miR-283-5p	AAAUAUCAGCUAGAUGCCUG ...........G.UAAUUCUGG	NW_017552654.1:190394..190458: −
asp-miR-125790-3p asp-miR-125790-5p	NW_017551561.1_12579	2.1	0.9	0	aca-miR-9573-3p	CAAAACUAGAAUUGCCUAGG .........G....U...U.	NW_017551561.1:47313..47366: +
	NW_017549024.1_10631	1.9	0.9	0			NW_017549024.1:233711..233804: +
asp-miR-56900-5p asp-miR-56900-3p	NW_017547229.1_5690	0.3	0.9	0	hsa-miR-483-5p	AUAAGACUGGAGGAAUGUAG .....C.......A.G...GAG	NW_017547229.1:2771708..2771774: +
asp-miR-77520-5p asp-miR-77520-3p	NW_017547298.1_7752	1.8	0.7	0	sfr-miR-10458-5p	CACUGGAUACAUGUUAAACG GC.G......G....U.C..	NW_017547298.1:3903205..3903268: +
asp-miR-147690-5p asp-miR-147690-3p	NW_017557621.1_14769	0	0.7	0	cre-miR1171	AGGAGUGGAUUUUCGAAC U........G.GGA.UGGAGUGG	NW_017557621.1:3670592..3670638: −
asp-miR-102140-5p asp-miR-102140-3p	NW_017548287.1_10214	0.5	0.5	0	ppc-miR-2263	GUCUCGGGUUCGAUUCCCG ........C.UCU..GAG.AU	NW_017548287.1:869888..869958: +
asp-miR-64380-3p asp-miR-64380-5p	NW_017547257.1_6438	0.7	0.4	0.4	spu-miR-4848a	AGGGUUGAGUAAGGACAUCAAC U........GCUUUUGGG..GGA	NW_017547257.1:3219088..3219147: +
asp-miR-134420-3p asp-miR-134420-5p	NW_017553232.1_13442	0.9	0.2	0	bta-miR-2489	AAGGGCUCAUGAGUUUUUG AAAUG.C....GA.........	NW_017553232.1:988520..988580: −
	NW_017553232.1_13405	0.9	0.2	0			NW_017553232.1:988522..988582: +
asp-miR-98810-5p asp-miR-98810-3p	NW_017548003.1_9881	0.4	0.2	0	efu-miR-9304	UAAGCCCCCCUCCCCUCCC G.......UUGAAGGG..AGGGG	NW_017548003.1:5351436..5351483: −
asp-miR-33140-5p asp-miR-33140-3p	NW_017547161.1_3314	0.1	0.2	0	bta-miR-2460	GAAACUCAUGAGCCCUGGC U..G...U....G......AU	NW_017547161.1:6988319..6988379: +

^a^ Non-significant randfold *p*-value. ^b^ Significant (e-value < 0.01) BLAST alignment between miRBase precursor and *Bemisia tabaci* genome.

## Supplementary Materials

The following are available online at https://www.mdpi.com/2075-4450/11/3/161/s1, Figure S1: Relationships between mature miRNAs and isomiRs of *Aleurocanthus spiniferus*. The isomiR diversity index is defined as the number of different isomiR sequences relative to the mature miRNA count (RPM). The isomiR abundance index is defined as the total isomiR count relative to the mature miRNA count (RPM). Table S1: Canonical miRNAs detected by miRDeep2 tool using *Aleurocanthus spiniferus* small RNA reads, *Bemisia tabaci* genome, and miRBase database. Table S2: Novel miRNAs (more than two mismatches shared with miRBase sequences) detected by miRDeep2 tool using *Aleurocanthus spiniferus* small RNA reads, *Bemisia tabaci* genome, and miRBase 22 database. Table S3: List and read count of mature miRNAs and isomiRs of *Aleurocanthus spiniferus*. Table S4: Overview of the isomiRs associated with the miRNAs of *Aleurocanthus spiniferus*.
