# Supplementary material for: First Report of Aleurocanthus spiniferus on Ailanthus altissima: Profiling of the Insect Microbiome and MicroRNAs
Source: Insects. 2020 Mar 3;11(3):161. doi: 10.3390/insects11030161 (PMC7142546; doi:10.3390/insects11030161)
Supplement: Supplementary file 1 [file insects-11-00161-s001.zip › insects-715338-supplementary/TabS4.pdf]

Table S4. Overview of the isomiRs associated with the miRNAs of *Aleurocanthus spiniferus*.

miRNA name	Feature	Example of perfect Match with miRBase miRNAs	miRNA/isomiR ID	Sequence	RPM	IsomiR diversity index	IsomiR abundance index
asp-bantam-3p	Mature miRNA		NW_017547202.1_5067	TGAGATCATCGTGAAAGCTG	33,695.0	0.02	2.43
	Most abundant isomiR		NW_017547202.1_5067.1	GAGATCATCGTGAAAGCTGA	2,054.2		
	isomiRs matching miRBase miRNAs	pca-bantam-3p	NW_017547202.1_5067.293	TGAGATCATTGTGAAAGCTGAT	12.7		
	Other isomiRs (606)				79,930.3		
asp-miR-276a-3p	Mature miRNA	aae-miR-276-3p	NW_017547107.1_1308	TAGGAACTTCATACCGTGCTC	10,088.2	0.03	0.77
	Most abundant isomiR		NW_017547107.1_1308.1	AGGAACTTCATACCGTGCTCT	27.5		
	isomiRs matching miRBase miRNAs	dme-miR-276a-3p	NW_017547107.1_1308.7	TAGGAACTTCATACCGTGCTCT	342.6		
		isc-miR-276	NW_017547107.1_1308.160	TAGGAACTTCATACCATGCTC	4.4		
	Other isomiRs (252)				7,362.8		
asp-miR-317-3p	Mature miRNA	dqu-miR-317-3p	NW_017550575.1_11879	TGAACACAGCTGGTGGTATCTCAG	811.3	0.29	3.81
	Most abundant isomiR		NW_017550575.1_11879.1	GAACACAGCTGGTGGTATCTCAGC	8.2		
	isomiRs matching miRBase miRNAs	ame-miR-317-3p	NW_017550575.1_11879.45	TGAACACAGCTGGTGGTATCTCAGT	8.4		
		api-miR-317	NW_017550575.1_11879.5	TGAACACAGCTGGTGGTATCT	233.3		
		mse-miR-317	NW_017550575.1_11879.4	TGAACACAGCTGGTGGTATCTCA	331.5		
	Other isomiRs (234)				2,509.4		
asp-miR-2765-5p	Mature miRNA		NW_017547098.1_410	TGGTAACTCCACACCACCGTTGG	1,212.5	0.10	0.88
	Most abundant isomiR		NW_017547098.1_410.1	GGTAACTCCACACCACCGTTGGC	15.6		
	isomiRs matching miRBase miRNAs	ame-miR-2765-5p	NW_017547098.1_412.2	TGGTAACTCCACCACCGTTGGC	143.1		
	Other isomiRs (186)				2,269.3		
asp-miR-750-3p	Mature miRNA	ame-miR-750-3p	NW_017547443.1_8884	CCAGATCTAACTCTTCCAGCTC	1,210.5	0.12	1.70
	Most abundant isomiR		NW_017547443.1_8884.1	CAGATCTAACTCTTCCAGCTCT	2.0		
	Other isomiRs (150)				2,060.2		
asp-miR-184-3p	Mature miRNA	aga-miR-184	NW_017547113.1_1681	TGGACGGAGAACTGATAAGGG	905.5	0.16	2.30
	Most abundant isomiR		NW_017547113.1_1681.1	GGACGGAGAACTGATAAGGGC	18.6		
	isomiRs matching miRBase miRNAs	dme-miR-184-3p	NW_017547113.1_1681.4	TGGACGGAGAACTGATAAGGGC	263.1		
		dqu-miR-184-3p	NW_017547113.1_1681.2	TGGACGGAGAACTGATAAGG	418.6		
		gga-miR-184-3p	NW_017547113.1_1681.27	TGGACGGAGAACTGATAAGGGT	14.0		
	Other isomiRs (140)				1,414.3		
asp-mir-8-3p	Mature miRNA		NW_017547115.1_2104	TAATACTGTCAGGTAATGAT	1,606.1	0.07	0.89
	Most abundant isomiR		NW_017547115.1_2104.1	AATACTGTCAGGTAATGATG	7.8		
	isomiRs matching miRBase miRNAs	api-miR-8	NW_017547115.1_2104.75	TAATACTGTCAGGTAATGATGTC	2.7		
		cqu-miR-8-3p	NW_017547115.1_2104.42	TAATACTGTCAGGTAAAGATGT	5.5		
	Other isomiRs (115)				1,409.8		
asp-miR-100-5p	Mature miRNA	tgu-miR-100-5p	NW_017547299.1_7860	AACCCGTAGATCCGAACTTGT	904.4	0.09	0.88
	Most abundant isomiR		NW_017547299.1_7860.1	ACCCGTAGATCCGAACTTGTG	4.7		
	isomiRs matching miRBase miRNAs	ame-miR-100-5p	NW_017547299.1_7860.5	AACCCGTAGATCCGAACTTGTG	43.5		
		ola-miR-100	NW_017547299.1_7860.3	AACCCGTAGATCCGAACTT	128.2		
	Other isomiRs (77)				622.0		
asp-miR-13a-3p	Mature miRNA		NW_017547352.1_8563	TATCACAGCCACTTTGATGAAC	926.6	0.08	0.69
	Most abundant isomiR		NW_017547352.1_8563.1	ATCACAGCCACTTTGATGAACT	1.5		
	Other isomiRs (71)				634.8		
asp-miR-2a-3p	Mature miRNA	mse-miR-2a	NW_017547352.1_8561	TCACAGCCAGCTTTGATGAGCA	155.7	0.64	4.43
	isomiRs matching miRBase miRNAs	api-miR-2a	NW_017561640.1_15257.6	TATCACAGCCAGCTTTGATGAGCG	39.5		

miRNA name	Feature	Example of perfect Match with miRBase miRNAs	miRNA/isomiR ID	Sequence	RPM	IsomiR diversity index	IsomiR abundance index
		dme-miR-2a-3p	NW_017547352.1_8561.6	TATCACAGCCAGCTTTGATGAGC	54.0		
		mse-miR-2b	NW_017547352.1_8561.7	TCACAGCCAGCTTTGATGAGC	49.6		
		pte-miR-2g-3p	NW_017547352.1_8561.1	TATCACAGCCAGCTTTGATGAG	47.8		
		pxy-miR-2b	NW_017547352.1_8561.4	TCACAGCCAGCTTTGATGAGCAC	155.3		
	Other isomiRs (173)				1,029.3		
asp-miR-315-5p	Mature miRNA	dme-miR-315-5p	NW_017547334.1_8451	TTTTGATTGTTGCTCAGAAAGC	447.9	0.21	2.18
	Most abundant isomiR		NW_017547334.1_8451.1	TTTGATTGTTGCTCAGAAAGCC	36.0		
	isomiRs matching miRBase miRNAs	dqu-miR-315-5p	NW_017547334.1_8451.42	TTTTGATTGTTGCTCAGAAAGCCT	3.3		
		dvi-miR-315a	NW_017547334.1_8451.2	TTTTGATTGTTGCTCAGAAAGCC	336.8		
		isc-miR-315	NW_017547334.1_8451.96	TTTTGATTGTTGCTCAGAAGGC	1.1		
		sko-miR-315	NW_017547334.1_8451.3	TTTTGATTGTTGCTCAGAAAG	97.1		
	Other isomiRs (90)				504.0		
asp-miR-9a-5p	Mature miRNA		NW_017551177.1_12283	TCTTTGGTTACCTAGCTGTATG	506.8	0.17	1.63
	Most abundant isomiR		NW_017551177.1_12283.1	CTTTGGTTACCTAGCTGTATGA	4.4		
	isomiRs matching miRBase miRNAs	aca-miR-9-5p	NW_017551177.1_12283.56	TCTTTGGTTATCTAGCTGTA	2.2		
		ppc-miR-9	NW_017551177.1_12283.36	TCTTTGGTTATCTAGCTGTATG	4.6		
		tca-miR-9a-5p	NW_017551177.1_12283.44	TCTTTGGTTATCTAGCTGTAT	3.5		
		xtr-miR-9b-5p	NW_017551177.1_12283.5	TCTTTGGTTACCTAGCTGTATGA	40.9		
	Other isomiRs (79)				772.6		
asp-miR-277-3p	Mature miRNA	aae-miR-277-3p	NW_017550575.1_11881	TAAATGCACTATCTGGTACGAC	486.2	0.12	1.09
	Most abundant isomiR		NW_017550575.1_11881.1	TAAATGCACTATCTGGTACGA	190.4		
	isomiRs matching miRBase miRNAs	dme-miR-277-3p	NW_017550575.1_11881.44	TAAATGCACTATCTGGTACGACA	1.6		
	Other isomiRs (57)				337.6		
asp-miR-71-3p	Mature miRNA		NW_017547352.1_8559	TCTCACTACCTTGTCTTTCAT	520.4	0.09	0.58
	Most abundant isomiR		NW_017547352.1_8559.1	CTCACTACCTTGTCTTTCATG	2.6		
	isomiRs matching miRBase miRNAs	tca-miR-71-3p	NW_017547352.1_8559.7	TCTCACTACCTTGTCTTTCATG	17.5		
	Other isomiRs (43)				283.3		
asp-miR-14-3p	Mature miRNA	dan-miR-14	NW_017547098.1_493	TCAGTCTTTTTCTCTCTCCTA	371.1	0.12	1.16
	Most abundant isomiR		NW_017547098.1_493.1	CAGTCTTTTTCTCTCTCCTAT	2.2		
	isomiRs matching miRBase miRNAs	dme-miR-14-3p	NW_017547098.1_493.4	TCAGTCTTTTTCTCTCTCCTAT	53.3		
	Other isomiRs (44)				376.6		
asp-miR-275-3p	Mature miRNA	api-miR-275	NW_017547297.1_7629	TCAGGTACCTGAAGTAGCGCG	276.4	0.20	1.80
	Most abundant isomiR		NW_017547297.1_7629.1	CAGGTACCTGAAGTAGCGCGC	4.4		
	isomiRs matching miRBase miRNAs	aae-miR-275-3p	NW_017547297.1_7629.3	TCAGGTACCTGAAGTAGCGC	88.2		
		dme-miR-275-3p	NW_017547297.1_7629.5	TCAGGTACCTGAAGTAGCGCGCG	35.1		
		tcf-miR-275	NW_017547297.1_7629.2	TCAGGTACCTGAAGTAGCGCGC	134.0		
	Other isomiRs (51)				234.9		
asp-let-7-5p	Mature miRNA	tca-let-7-5p	NW_017547299.1_7862	TGAGGTAGTAGGTTGTATAG	257.8	0.19	1.70
	Most abundant isomiR		NW_017547299.1_7862.1	GAGGTAGTAGGTTGTATAGT	13.1		
	isomiRs matching miRBase miRNAs	dme-let-7-5p	NW_017547299.1_7862.3	TGAGGTAGTAGGTTGTATAGT	153.7		
		hme-let-7	NW_017547299.1_7862.5	TGAGGTAGTAGGTTGTATAGTA	38.7		
		pte-let-7-5p	NW_017547299.1_7862.17	TGAGGTAGTAGGTTGTATAGTT	4.0		
	Other isomiRs (45)				227.8		
asp-miR-263a-5p	Mature miRNA		NW_017547161.1_3192	AATGGCACTAGAAGAATTCACG	173.5	0.41	2.72
	Most abundant isomiR		NW_017547161.1_3192.1	ATGGCACTAGAAGAATTCACGG	7.5		
	isomiRs matching miRBase miRNAs	aae-miR-263a-5p	NW_017557870.1_14945.2	AATGGCACTGGAAGAATTCACGG	4.4		

miRNA name	Feature	Example of perfect Match with miRBase miRNAs	miRNA/isomiR ID	Sequence	RPM	IsomiR diversity index	IsomiR abundance index
		bmo-miR-263a-5p	NW_017557870.1_14945.1	AATGGCACTGGAAGAATTCAC	8.6		
		dme-miR-263a-5p	NW_017557870.1_14945.5	AATGGCACTGGAAGAATTCACGGG	2.7		
		tur-miR-263a-5p	NW_017557870.1_14945.4	TATGGCACTGGAAGAATTCACG	3.5		
	Other isomiRs (71)				488.8		
asp-miR-31-5p	Mature miRNA		NW_017547107.1_1345	AGGCAAGAUGUUGGCAUAGC	450.0	0.20	2.16
	Most abundant isomiR	aca-miR-31-5p	NW_017547107.1_1345.1	AGGCAAGAUGUUGGCAUAGCC	30.0		
	Other isomiRs (90)				174.4		
asp-miR-306-5p	Mature miRNA		NW_017547320.1_8138	TCAGGTACTGAGTGACTCTG	192.9	0.24	1.88
	Most abundant isomiR		NW_017547320.1_8138.1	CAGGTACTGAGTGACTCTGA	3.5		
	isomiRs matching miRBase miRNAs	ame-miR-306-5p	NW_017547320.1_8138.3	TCAGGTACTGAGTGACTCTGAG	118.4		
		dqu-miR-306-5p	NW_017547320.1_8138.4	TCAGGTACTGAGTGACTCTGA	54.0		
	Other isomiRs (44)				186.2		
asp-miR-92b-3p	Mature miRNA		NW_017564582.1_15992	AAUUGCACUAGUCCCGGCCUG	150.0	0.04	2.26
	isomiRs matching miRBase miRNAs	dme-miR-92b-3p	NW_017564582.1_15992.1	AAUUGCACUAGUCCCGGCCUGC	16.2		
	Other isomiRs (6)				322.2		
asp-miR-305-5p	Mature miRNA	dvi-miR-305-5p	NW_017547297.1_7632	ATTGTACTTCATCAGGTGCTC	99.1	0.60	3.67
	Most abundant isomiR		NW_017547297.1_7632.1	TTGTACTTCATCAGGTGCTCT	3.5		
	isomiRs matching miRBase miRNAs	aae-miR-305-5p	NW_017547297.1_7632.6	ATTGTACTTCATCAGGTGCTCTGG	21.6		
		cqu-miR-305-5p	NW_017547297.1_7632.7	ATTGTACTTCATCAGGTGCTCT	21.6		
		dme-miR-305-5p	NW_017547297.1_7632.4	ATTGTACTTCATCAGGTGCTCTG	91.8		
		pca-miR-305-5p	NW_017547297.1_7632.5	ATTGTACTTCATCAGGTGCT	39.1		
	Other isomiRs (54)				185.7		
asp-miR-87a-3p	Mature miRNA		NW_017547298.1_7719	GTGAGCAACGTATCAGGTGTC	61.1	0.13	0.47
	Most abundant isomiR		NW_017547298.1_7719.1	AGCAACGTATCAGGTGTC	1.1		
	isomiRs matching miRBase miRNAs	ame-miR-87-3p	NW_017547298.1_7717.5	GTGAGCAAAGTTTCAGGTGT	3.8		
		dqu-miR-87-3p	NW_017547298.1_7717.9	GTGAGCAAAGTTTCAGGTGTGT	1.8		
	Other isomiRs (27)				286.9		
asp-miR-13b-3p	Mature miRNA		NW_017561640.1_15253	TATCACAGCCATTTTTGACGTGCC	80.0	0.54	2.41
	Most abundant isomiR		NW_017561640.1_15253.1	TATCACAGCCATTTTTGACGTGC	50.2		
	Other isomiRs (42)				142.4		
asp-miR-34-5p	Mature miRNA	bmo-miR-34-5p	NW_017550575.1_11884	TGGCAGTGTGGTTAGCTGGTTG	66.2	0.42	2.77
	Most abundant isomiR		NW_017550575.1_11884.1	GGCAGTGTGGTTAGCTGGTTGT	4.4		
	isomiRs matching miRBase miRNAs	aga-miR-34	NW_017550575.1_11884.3	TGGCAGTGTGGTTAGCTGGT	43.6		
		ame-miR-34-5p	NW_017550575.1_11884.35	TGGCAGTGTTGTTAGCTGGTTG	0.9		
		dme-miR-34-5p	NW_017550575.1_11884.6	TGGCAGTGTGGTTAGCTGGTTGTG	7.3		
		dvi-miR-34-5p	NW_017550575.1_11884.2	TGGCAGTGTGGTTAGCTGGTT	43.8		
		mse-miR-34	NW_017550575.1_11884.4	TGGCAGTGTGGTTAGCTGGTTGT	32.4		
		ngi-miR-34	NW_017550575.1_11884.9	GGCAGTGTGGTTAGCTGGTTG	4.6		
	Other isomiRs (21)				46.4		
asp-miR-993-3p	Mature miRNA		NW_017547290.1_7299	GAAGCTCGTCTCTACAGGTATC	152.6	0.18	0.63
	Most abundant isomiR		NW_017547290.1_7299.1	GAAGCTCGTCTCTACAGGTAT	31.6		
	isomiRs matching miRBase miRNAs	dme-miR-993-3p	NW_017547290.1_7299.6	GAAGCTCGTCTCTACAGGTATCT	4.7		
	Other isomiRs (25)				59.8		
asp-miR-125-5p	Mature miRNA		NW_017547299.1_7864	TCCCTGAGACCCTAATTTGTG	142.0	0.13	0.72
	Most abundant isomiR		NW_017547299.1_7864.1	CCCTGAGACCCTAATTTGTGA	1.1		
	Other isomiRs (17)				101.7		

miRNA name	Feature	Example of perfect Match with miRBase miRNAs	miRNA/isomiR ID	Sequence	RPM	IsomiR diversity index	IsomiR abundance index
asp-miR-927-3p	Mature miRNA		NW_017547282.1_7013	CAAAGCGTTTGAATTCTGGAA	145.8	0.10	0.42
	Most abundant isomiR		NW_017547282.1_7013.1	CAAAGCGTTTGAATTCTGGAAC	17.5		
	Other isomiRs (13)				43.3		
asp-miR-281-2-5p	Mature miRNA		NW_017561619.1_15248	AAGAGAGCTATCCTTCGACAG	71.6	0.38	1.64
	Most abundant isomiR		NW_017561619.1_15248.1	AGAGAGCTATCCTTCGACAGT	7.3		
	isomiRs matching miRBase miRNAs	aae-miR-281-5p	NW_017561619.1_15248.15	AAGAGAGCTATCCGTCGAC	2.7		
		dme-miR-281-2-5p	NW_017561619.1_15248.3	AAGAGAGCTATCCGTCGACAGT	14.0		
	Other isomiRs (24)				93.6		
asp-miR-252a-5p	Mature miRNA		NW_017554870.1_13884	CTAAGTACTCCGTGCCGCAGG	70.6	0.30	1.63
	Most abundant isomiR		NW_017554870.1_13884.1	TAAGTACTCCGTGCCGCAGGA	7.5		
	Other isomiRs (20)				107.6		
asp-miR-1-3p	Mature miRNA		NW_017547904.1_9568	TGGAATGTAAAGAAGTATGGA	62.0	0.26	0.87
	Most abundant isomiR		NW_017547904.1_9568.1	TGGAATGTAAAGAAGTATGG	10.7		
	isomiRs matching miRBase miRNAs	cbr-miR-1	NW_017547904.1_9568.18	TGGAATGTAAAGAAGTATGTA	1.1		
		dme-miR-1-3p	NW_017547904.1_9568.4	TGGAATGTAAAGAAGTATGGAG	6.7		
	Other isomiRs (13)				35.6		
asp-miR-190-5p	Mature miRNA	bmo-miR-190-5p	NW_017547096.1_359	AGATATGTTTGATATTCTTGGTT	42.9	0.33	1.18
	Most abundant isomiR		NW_017547096.1_359.1	GATATGTTTGATATTCTTGGTTG	1.1		
	isomiRs matching miRBase miRNAs	dme-miR-190-5p	NW_017547096.1_359.3	AGATATGTTTGATATTCTTGGTTG	10.2		
		hme-miR-190	NW_017547096.1_359.2	AGATATGTTTGATATTCTTGGT	10.9		
		tca-miR-190-5p	NW_017547096.1_359.6	AGATATGTTTGATATTCTTGG	3.8		
	Other isomiRs (10)				24.7		
asp-miR-133-3p	Mature miRNA	cin-miR-133-3p	NW_017547904.1_9564	TTGGTCCCCTTCAACCAGCTG	35.6	0.22	0.93
	isomiRs matching miRBase miRNAs	dme-miR-133-3p	NW_017547904.1_9564.4	TTGGTCCCCTTCAACCAGCTGT	3.5		
		pal-miR-133b-3p	NW_017547904.1_9564.1	TTGGTCCCCTTCAACCAGCT	14.6		
	Other isomiRs (6)				15.1		
asp-miR-92a-3p	Mature miRNA		NW_017564582.1_15994	TATTGCACTTGTCCCGGCC	21.5	0.51	2.13
	isomiRs matching miRBase miRNAs	aae-miR-92a-3p	NW_017564582.1_15994.1	TATTGCACTTGTCCCGGCCTA	16.2		
		aga-miR-92a	NW_017564582.1_15994.3	TATTGCACTTGTCCCGGCCTAT	3.3		
		bma-miR-92	NW_017564582.1_15994.5	TATTGCACTCGTCCCGGCCT	2.6		
		cpi-miR-92a-3p	NW_017564582.1_15994.2	TATTGCACTTGTCCCGGCCT	11.3		
		sha-miR-92a	NW_017564582.1_15994.4	TATTGCACTTGTCCCGGC	2.7		
	Other isomiRs (6)				9.6		
asp-miR-263b-5p	Mature miRNA		NW_017547161.1_3196	CTTGGCACTGGAAGAATTCACAG	18.2	0.77	2.28
	isomiRs matching miRBase miRNAs	ame-miR-263b-5p	NW_017547161.1_3196.1	CTTGGCACTGGAAGAATTCAC	12.6		
		api-miR-263b	NW_017547161.1_3196.3	CTTGGCACTGGAAGAATTCACAGA	2.9		
		dpu-miR-263b	NW_017547161.1_3196.2	CTTGGCACTGGAAGAATTCACA	9.5		
		nvi-miR-263b	NW_017547161.1_3196.10	TTGGCACTGGAAGAATTCAC	1.3		
	Other isomiRs (10)				15.3		
asp-miR-252b-5p	Mature miRNA		NW_017554870.1_13882	CTAAGTAGTAGCGCCGAAGGTG	19.6	0.71	1.94
	Most abundant isomiR		NW_017554870.1_13882.1	CTAAGTAGTAGCGCCGAAGG	9.6		
	Other isomiRs (13)				28.4		
asp-miR-124-3p	Mature miRNA	pca-miR-124-3p	NW_017557672.1_14877	TAAGGCACGCGGTGAATGCC	30.7	0.23	0.80
	isomiRs matching miRBase miRNAs	aae-miR-124	NW_017557672.1_14877.1	TAAGGCACGCGGTGAATGC	10.4		
		api-miR-124	NW_017557672.1_14877.3	TAAGGCACGCGGTGAATGCCA	3.5		
		hme-miR-124	NW_017557672.1_14877.5	TAAGGCACGCGGTGAATGCCAA	1.8		

miRNA name	Feature	Example of perfect Match with miRBase miRNAs	miRNA/isomiR ID	Sequence	RPM	IsomiR diversity index	IsomiR abundance index
	Other isomiRs (4)				8.9		
asp-miR-137-3p	Mature miRNA		NW_017547181.1_4201	TATTGCTTGAGAATACACGTA	13.1	0.23	0.33
asp-miR-375-3p	Mature miRNA		NW_017547090.1_76	TTTGTTCGTTCGGCTCGAGTT	16.7	0.36	1.38
	Most abundant isomiR		NW_017547090.1_76.1	TTTGTTCGTTCGGCTCGAGT	6.7		
	isomiRs matching miRBase miRNAs	ame-miR-375-3p	NW_017547090.1_76.3	TTTGTTCGTTCGGCTCGAGTTA	4.2		
	Other isomiRs (4)				12.2		
asp-miR-10-5p	Mature miRNA		NW_017547290.1_7361	TACCCTGTAGATCCGAAATT	22.0	0.18	1.35
	Most abundant isomiR	oar-miR-10a	NW_017547290.1_7361.1	TACCCTGTAGATCCGTAATG	6.0		
	Other isomiRs (4)				23.8		
asp-miR-996-3p	Mature miRNA		NW_017553907.1_13657	TGACTAGAGTTACACTCGTC	15.6	0.38	1.45
	Most abundant isomiR		NW_017553907.1_13657.1	TGACTAGAGTTACACTCGT	8.7		
	Other isomiRs (5)				14.0		
asp-miR-3049-3p	Mature miRNA		NW_017547142.1_3035	TCCGTCCAACTCTTTTCCGCC	12.9	0.00	0.00
asp-miR-210-3p	Mature miRNA		NW_017550873.1_12017	CTTGTGCGTGTGACAGCGGC	11.5	0.61	1.44
	isomiRs matching miRBase miRNAs	ame-miR-210-3p	NW_017550873.1_12017.6	TTGTGCGTGTGACAGCGGCTA	1.6		
		bdo-miR-210	NW_017550873.1_12017.8	TTGTGCGTGTGACAGCGGCTAT	1.3		
		bmo-miR-210	NW_017550873.1_12017.1	TTGTGCGTGTGACAGCGGCT	2.9		
		sfr-miR-210-3p	NW_017550873.1_12017.3	CTTGTGCGTGTGACAGCGGCT	2.6		
	Other isomiRs (3)				8.2		
asp-miR-7-5p	Mature miRNA	dme-miR-7-5p	NW_017556795.1_14461	TGGAAGACTAGTGATTTTGTTGT	7.8	0.90	2.44
	isomiRs matching miRBase miRNAs	dvi-miR-7-5p	NW_017556795.1_14461.5	TGGAAGACTAGTGATTTTGTTGTT	1.5		
		hme-miR-7	NW_017556795.1_14461.1	TGGAAGACTAGTGATTTTGTTG	6.2		
		sfr-miR-7-5p	NW_017556795.1_14461.3	TGGAAGACTAGTGATTTTGTT	2.2		
	Other isomiRs (4)				9.3		
asp-miR-1000-5p	Mature miRNA		NW_017547246.1_6234	ATATTGTCCTGTCACAGCAG	9.5	0.53	1.82
	isomiRs matching miRBase miRNAs	dme-miR-1000-5p	NW_017547246.1_6234.1	ATATTGTCCTGTCACAGCAGT	9.3		
		dps-miR-1000-5p	NW_017547246.1_6234.5	ATATTGTCCTGTCACAGCAGTA	1.5		
	Other isomiRs (3)				6.5		
asp-miR-929-5p	Mature miRNA	api-miR-929	NW_017547330.1_8238	AAATTGACTCTAGTAGGGAG	12.2	0.25	0.66
	Most abundant isomiR		NW_017547330.1_8238.1	AAATTGACTCTAGTAGGGA	6.0		
	isomiRs matching miRBase miRNAs	ame-miR-137-3p	NW_017547181.1_4201.3	TTATTGCTTGAGAATACACGTA	1.5		
	Other isomiRs (2)				3.5		
asp-miR-29b-3p	Mature miRNA	tgu-miR-29b-3p	NW_017547261.1_6573	TAGCACCATTTGAAATCAGTG	6.7	0.59	1.81
	Most abundant isomiR		NW_017547261.1_6573.1	TAGCACCATTTGAAATCAG	5.6		
	isomiRs matching miRBase miRNAs	ame-miR-29b-3p	NW_017547261.1_6573.2	TAGCACCATTTGAAATCAGT	4.2		
	Other isomiRs (2)				2.4		
asp-miR-971b-5p	Mature miRNA		NW_017547177.1_3772	CACTCTAAGCTCGAACATCAAG	11.6	0.17	0.20
	Most abundant isomiR		NW_017547177.1_3772.1	CACTCTCAGCTCGAACATCAAG	1.5		
	Other isomiRs (1)				0.9		
asp-miR-307a-3p	Mature miRNA	dan-miR-307	NW_017547096.1_364	TCACAACCTCCTTGAGTGAG	4.7	1.06	1.85
	Most abundant isomiR		NW_017547096.1_364.1	CACAACCTCCTTGAGTGAGC	1.6		
	isomiRs matching miRBase miRNAs	dvi-miR-307	NW_017547096.1_364.4	TCACAACCTCCTTGAGTGAGC	1.6		
	Other isomiRs (3)				5.5		
asp-miR-2796-3p	Mature miRNA		NW_017547276.1_6782	GTAGGCCGGCGGAAACTACTTG	4.7	0.63	1.04
	Most abundant isomiR		NW_017547276.1_6782.1	GTAGGCCGGCGGAAACTACTT	2.0		
	isomiRs matching miRBase miRNAs	ame-miR-2796-3p	NW_017547276.1_6782.2	GTAGGCCGGCGGAAACTACTTGC	1.6		

miRNA name	Feature	Example of perfect Match with miRBase miRNAs	miRNA/isomiR ID	Sequence	RPM	IsomiR diversity index	IsomiR abundance index
	Other isomiRs (1)				1.3		
asp-miR-iab-4-5p	Mature miRNA		NW_017549609.1_11445	ACGTATACTAAATGTATCCT	2.6	0	0
	isomiRs matching miRBase miRNAs	dme-miR-965-3p	NW_017551483.1_12566.2	TAAGCGTATAGCTTTTCCCCTT	0.9		
		dvi-miR-965-3p	NW_017551483.1_12566.1	TAAGCGTATAGCTTTTCCCCT	1.2		
asp-miR-965-3p	Mature miRNA		NW_017551483.1_12566	TAAGCGTATAGCTTTTCCCC	4.7	0.42	0.62
asp-miR-iab-8-5p	Mature miRNA		NW_017549609.1_11368	AGGAUACAUUUAGUAUACGUC	0.4	0.77	0.46
