# Supplementary figures and images for: First Report of Aleurocanthus spiniferus on Ailanthus altissima: Profiling of the Insect Microbiome and MicroRNAs
Source: Insects. 2020 Mar 3;11(3):161. doi: 10.3390/insects11030161 (PMC7142546; doi:10.3390/insects11030161)

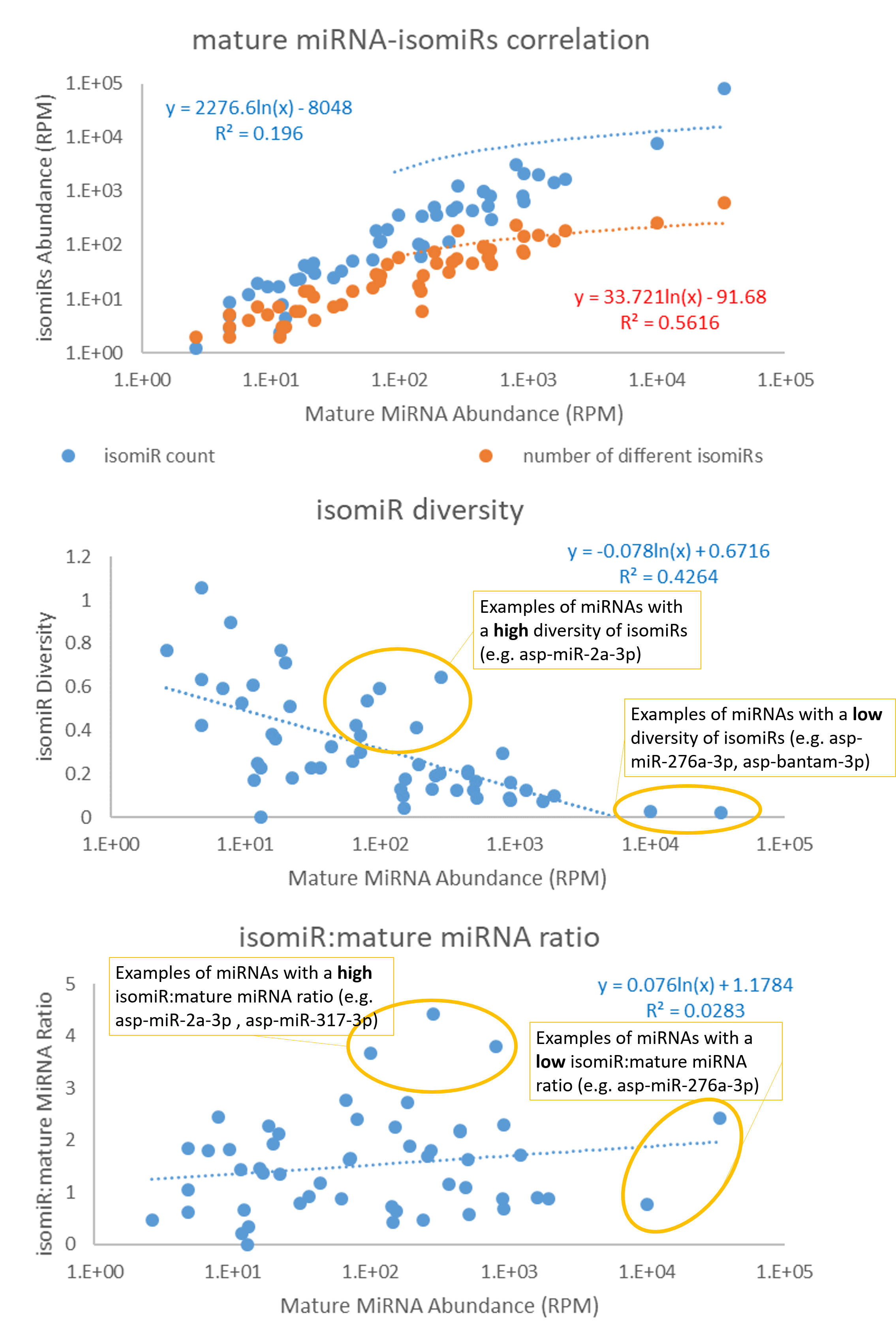

Supplement: Supplementary file 1 [file insects-11-00161-s001.zip › insects-715338-supplementary/FigS1.tif]
